# Total Synthesis and Biological Activities of Polyhydroxy Flavonols: A Review

**DOI:** 10.3390/molecules31122107

**Published:** 2026-06-15

**Authors:** Jia-Yao Liu, Jie Tao, Jing-Min Chen, Jia Li, Xin Meng, Xu-Dong Zhou, Cai-Yun Peng, Wen-Bing Sheng

**Affiliations:** 1 School of Pharmacy, Hunan University of Chinese Medicine, Changsha 410208, China; 20243725@stu.hnucm.edu.cn (J.-Y.L.); 20243726@stu.hnucm.edu.cn (J.T.); ace666@stu.hnucm.edu.cn (J.-M.C.); lijia424@stu.hnucm.edu.cn (J.L.); mx912@stu.hnucm.edu.cn (X.M.); xudongzhou999@hnucm.edu.cn (X.-D.Z.); 2 TCM and Ethnomedicine Innovation and Development International Laboratory, Hunan University of Chinese Medicine, Changsha 410208, China

**Keywords:** polyhydroxy flavonols, total synthesis, biological activity

## Abstract

Flavonols are an important class of flavonoids widely distributed across various plant species. They have garnered significant attention from synthetic chemists due to their extensive biological activities and medicinal value. This review provides a systematic overview of five classical synthetic methods, including the Auwers reaction, the Allan–Robinson reaction, the Baker–Venkataraman rearrangement, the Algar–Flynn–Oyamada (AFO) reaction, and DMDO-mediated oxidation. Each methodology is comprehensively discussed in terms of its advantages, limitations, and potential optimization strategies. Additionally, the biological activities, including antioxidant, anticancer, anti-inflammatory, and antiviral properties, are summarized and discussed in the context of structure–activity relationships (SARs).

## 1. Introduction

**Figure 1:**
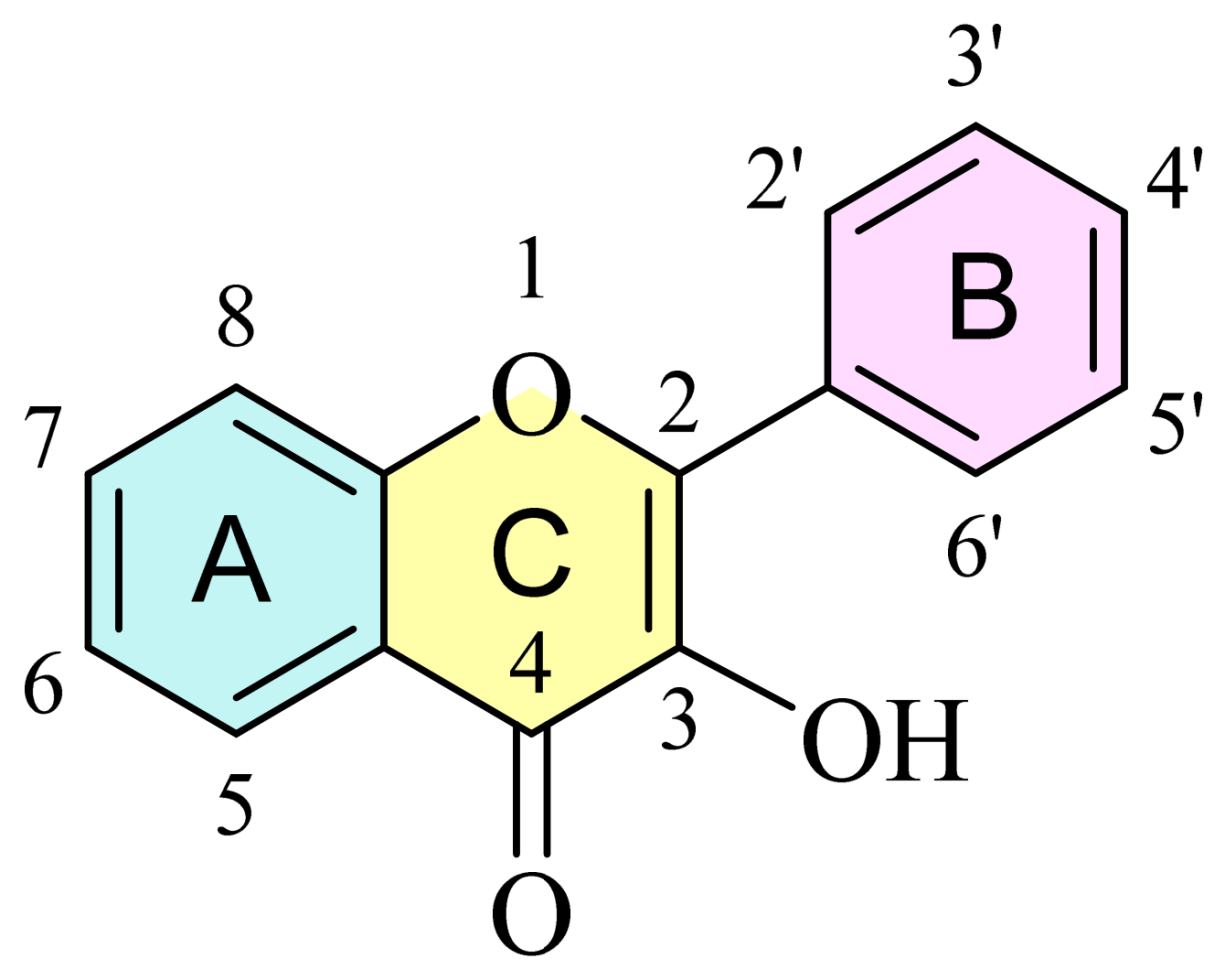
General structure of the flavonol scaffold (2-phenylchroman-4-one-3-ol) illustrating the C6-C3-C6 skeleton.

Flavonols represent a significant subclass of flavonoids, characterized by a core structure of 2-phenylchroman-4-one-3-ol with the typical C6-C3-C6 skeleton [1]. As significant secondary metabolites in plants, they are widely distributed in fruits, vegetables, and beverages [2]. The fundamental skeletal framework is shown in Figure 1, and representative compounds are depicted in Table 1.

Flavonols play diverse physiological roles in plants. Beyond their well-established functions in pigmentation, such as acting as co-pigments with anthocyanins [3,4], they serve as UV protectants, modulators of auxin transport for growth regulation, and defense compounds against herbivores and pathogens [5,6,7,8]. Due to their unique structural features, flavonols exhibit significant biological activities including antioxidant [9,10,11,12], anti-inflammatory [13,14,15,16], anticancer [17,18,19,20], antiviral [21,22,23,24], and anti-bacterial effects [25,26]. Consequently, these compounds hold extensive application prospects in the food [27,28], pharmaceutical [29,30], and cosmetic industries [31,32].

In recent years, flavonols and their derivatives have attracted increasing attention due to their novel structures and excellent pharmacological activities. However, systematic summaries of their total synthesis methods remain limited. The synthetic challenges primarily stem from regioselective hydroxylation [33], C3 oxidation state control [34], and susceptibility to oxidative degradation [35,36]. To address these challenges, several strategies have been developed, including the AFO reaction, the Baker–Venkataraman rearrangement, and the DMDO oxidation, while earlier approaches such as the Auwers and Allan–Robinson reactions have also contributed to the field. This review summarizes research progress in the total synthesis of flavonols, systematically examining these methodologies with emphasis on recent advances in efficiency, selectivity, and scalability. The SAR of polyhydroxy flavonols is also explored, aiming to provide references for structural optimization and drug development.

## 2. Total Synthetic Methods of Flavonols

### 2.1. Auwers Reaction

**Scheme 1:**
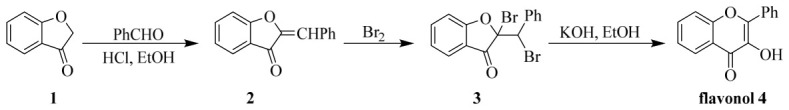
Synthetic route of flavonol **4** via the Auwers method.

The Auwers reaction was first reported by Karl von Auwers in 1908, in this reaction benzofuran-3-one **1** undergoes an aldol condensation reaction with benzaldehyde, followed by bromination and rearrangement under alkaline conditions to yield flavonol **4** (Scheme 1) [37,38].

This reaction has certain application value in the synthesis of flavonols. However, it imposes strict requirements on the structure of the reactants. Furthermore, the preparation of the dibromide intermediate is challenging, which often results in low overall yields and difficulties in scaling up production. Consequently, its application in synthesis remains limited.

### 2.2. Allan–Robinson Reaction

**Scheme 2:**
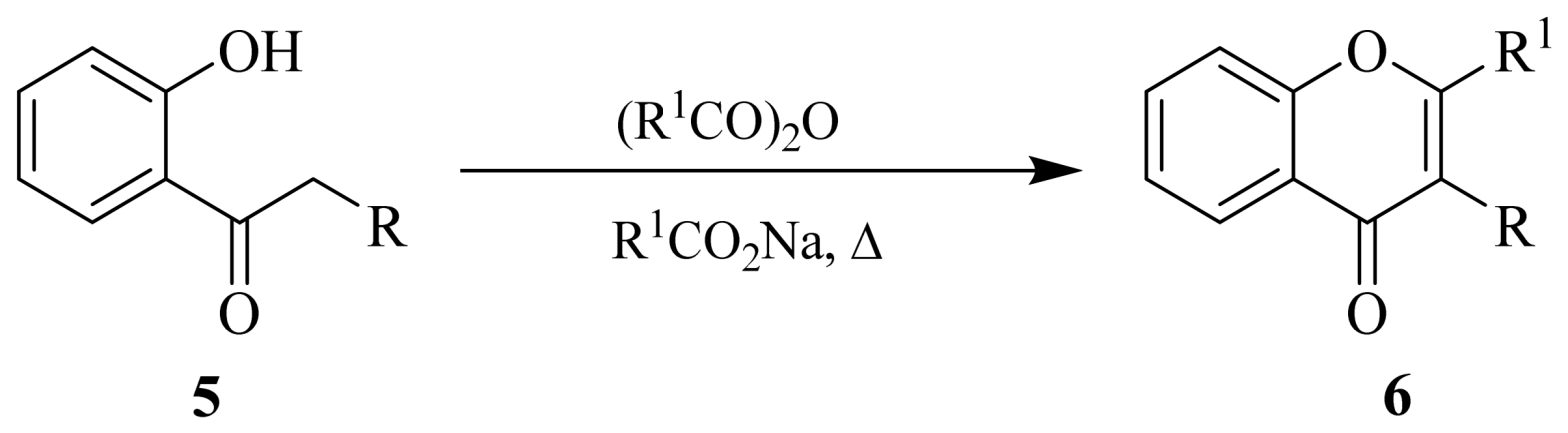
Synthetic route of flavonol **6** via the Allan–Robinson method.

Allan–Robinson reaction was reported by Allan and Robinson in 1924 [39]. A typical representative reaction involves the synthesis of flavonol **6** through intramolecular condensation of a O-hydroxyaryl ketone **5** and an aromatic anhydride under alkaline conditions (Scheme 2). When aliphatic anhydrides are used as the reactant, it is termed the Kostanecki reaction.

**Scheme 3:**
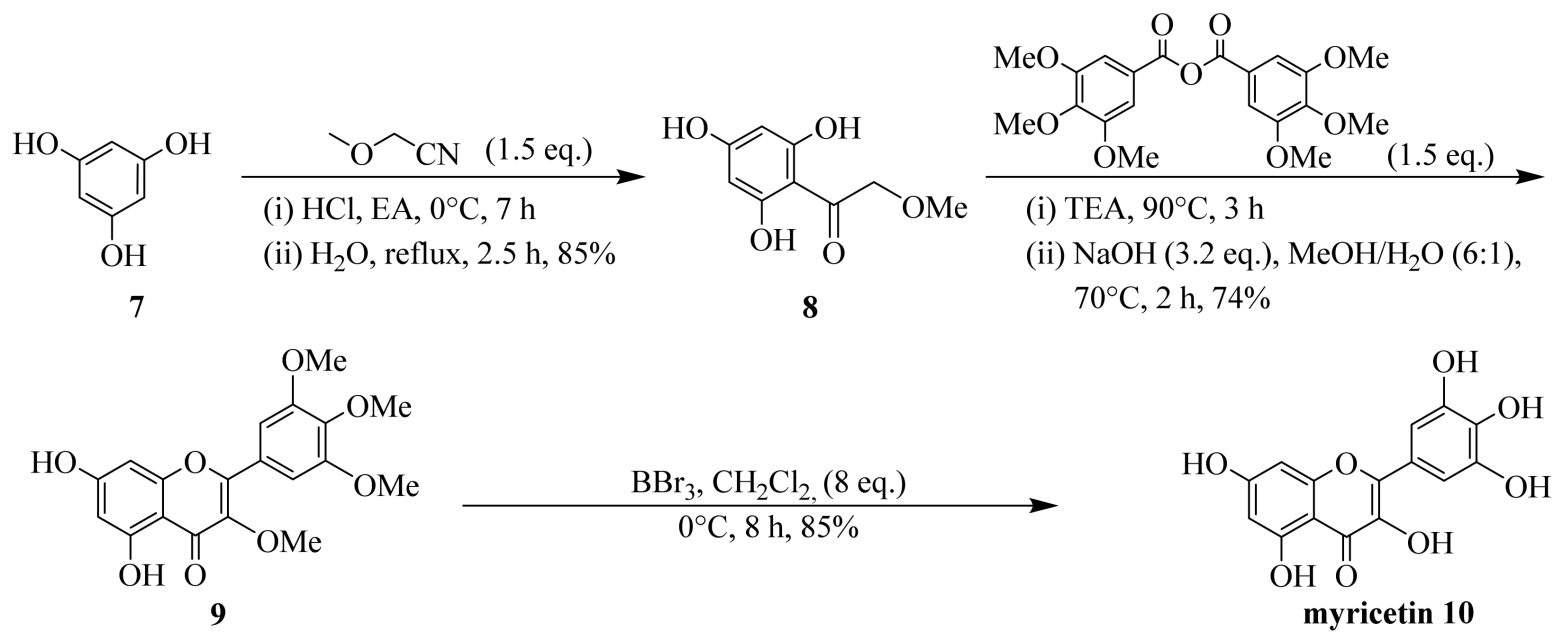
Synthetic route of myricetin **10**.

In 2024, Gu [40] improved the reaction conditions of the Allan–Robinson reaction. In her reaction the starting material was phloroglucinol **7**, it underwent the Houben-Hoesch reaction with methoxyacetonitrile to form hydroxyaryl ketone **8**. Intermediate **8** was then reacted with 3,4,5-trimethoxybenzoic anhydride via the Allan–Robinson reaction to yield **9**. After demethylation with BBr_3_/CH_2_Cl_2_, myricetin **10** was successfully synthesized (Scheme 3). This improved method allows the product to be purified through simple recrystallization, and achieves a total yield of up to 53%, demonstrating good potential for large-scale production. These characteristics significantly enhance the practicality and feasibility of the Allan–Robinson reaction.

Despite the synthetic advantages of the Allan–Robinson reaction as a one-pot process for esterification, rearrangement, and condensation, it suffers from prolonged reaction times, generally low yields, and the frequent formation of 3-aryloxyflavones [41]. Moreover, when symmetric aromatic anhydrides are used, atom economy is poor: only one aryl unit is incorporated into the flavonoid skeleton; the other is lost as carboxylic acid.

### 2.3. Baker–Venkataraman Rearrangement Reaction

**Scheme 4:**
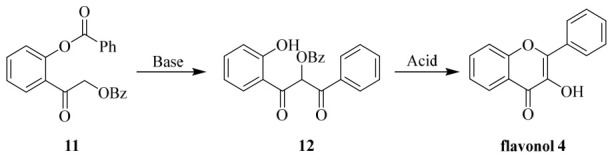
Synthetic route of flavonol **4** via the Baker–Venkataraman rearrangement.

The Baker–Venkataraman rearrangement, developed as a refined alternative to the Allan–Robinson condensation, is a widely employed method for the synthesis of flavonols [42]. In this process, compound **11** undergoes intramolecular Claisen condensation to form the corresponding intermediate *β*-diketone **12**, which is then cyclized under acid catalysis to yield flavonol **4** (Scheme 4). Notably, the *β*-diketone serves as a crucial intermediate in the Baker–Venkataraman rearrangement.

**Scheme 5:**
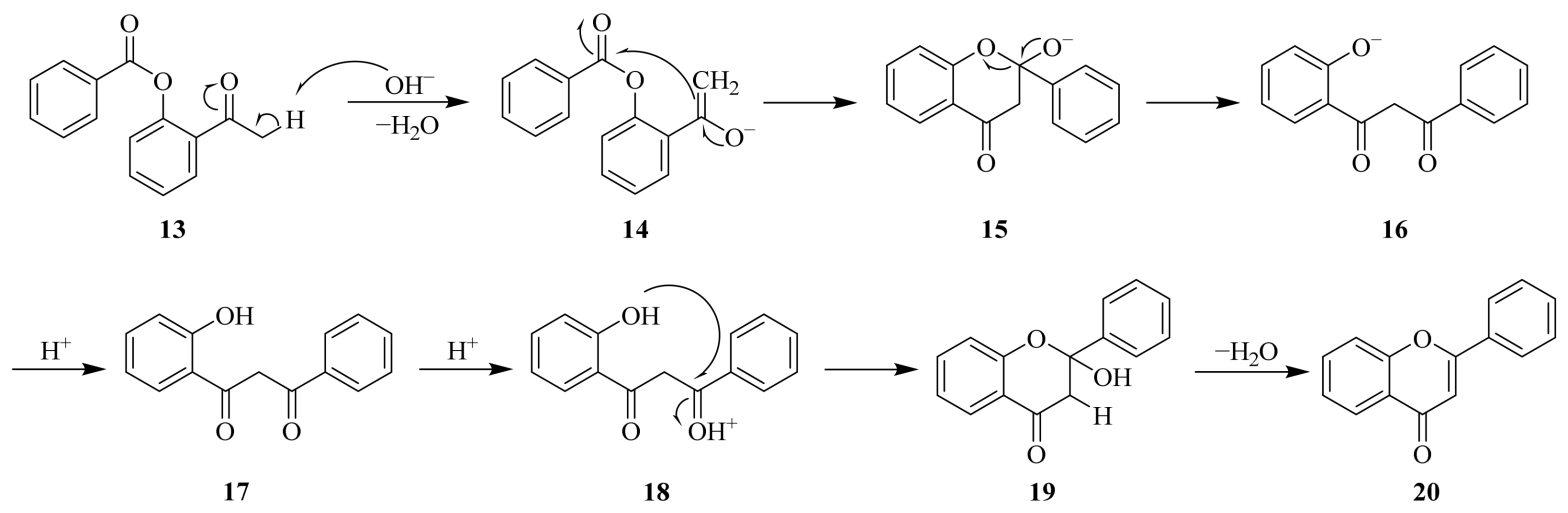
The mechanism of Baker–Venkataraman rearrangement reaction.

The Baker–Venkataraman rearrangement enables the construction of the flavonoid backbone through intramolecular acyl migration via a well-defined mechanistic pathway. The reaction is initiated by deprotonation of the o-hydroxyl group in o-acyloxyacetophenone **13** under strong base conditions, generating the enolate **14**, which undergoes intramolecular nucleophilic attack on the ester carbonyl to afford tetrahedral intermediate **15**. In this step, the acyl group migrates to the oxygen of the former o hydroxyl, furnishing 1,3-diketone **16**. Under acid catalysis, **16** undergoes protonation to give **17**, which tautomerizes to enol **18**. The enol then engages in an intramolecular nucleophilic attack, wherein the enolic hydroxyl attacks the ketone carbonyl carbon, leading to cyclic hemiketal intermediate **19**. Finally, dehydration of the hemiketal intermediate **19** affords the flavonoid **20** (Scheme 5). Subsequent oxidation or hydroxylation can further convert it into flavonol [43].

Commonly used bases for the Baker–Venkataraman rearrangement include KOH, NaOH, LiHMDS, K_2_CO_3_, pyridine, TEA, and DBU, while the cyclization step is typically promoted by H_2_SO_4_, AcOH/H_2_SO_4_, *p*-TsOH, TFA, PPA, or Lewis acids [44,45]. It is also essential to design selective protection and deprotection steps for the preparation of flavonols. Commonly protecting groups such as MeOCH_2_–, CH_3_CO–, or CH_3_O– exhibits stability under reaction conditions and can be efficiently removed in subsequent steps, thereby enabling high-yield synthesis of flavonols.

#### 2.3.1. Synthesis of Polyhydroxy Flavonols **24**

**Scheme 6:**
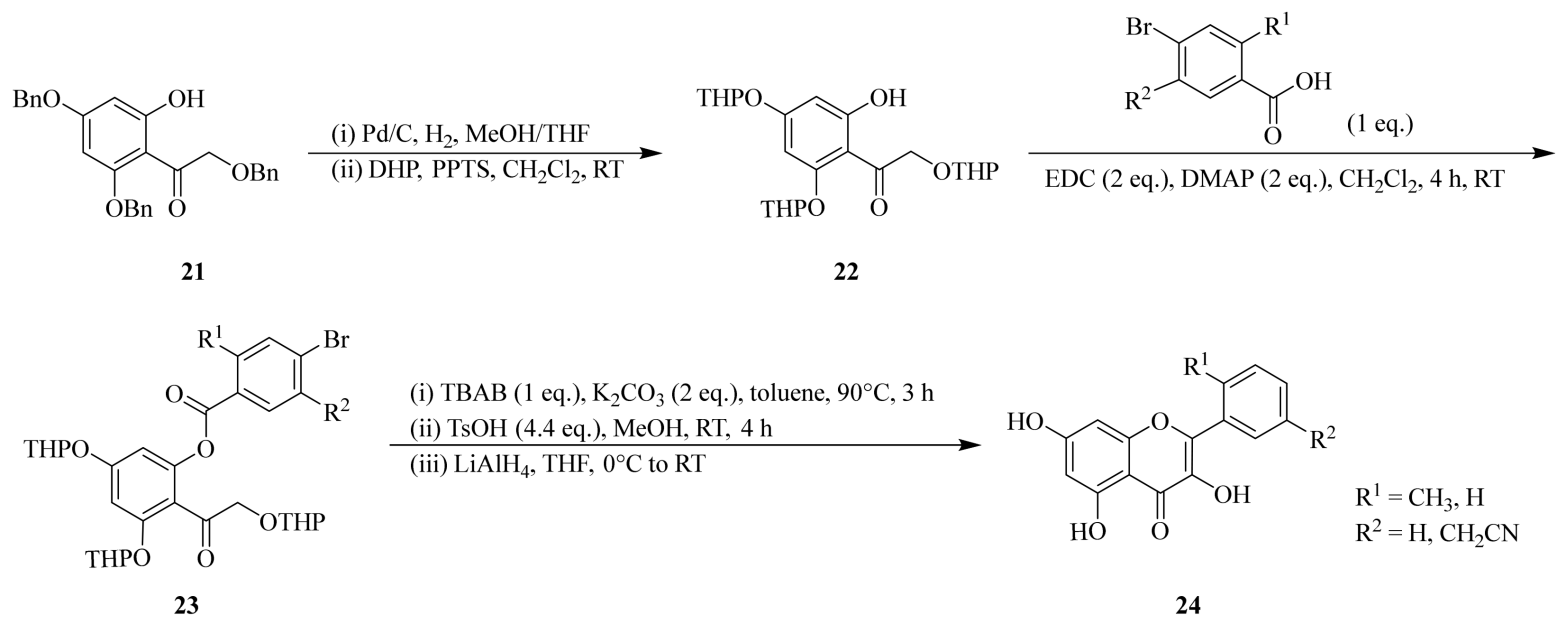
Synthetic route of polyhydroxy flavonols **24**.

Despite its reliability, the Baker–Venkataraman rearrangement involves three steps, esterification, rearrangement, and ring closure, rendering the process cumbersome. To streamline the synthesis, Kim et al. [46] developed a concise approach to prepare flavonol derivatives as selective JAK1 inhibitors in 2014. In this method, **22** was prepared from **21** by debenzylation followed by THP protection, and **21** was obtained by base-promoted fragmentation of perbenzylated quercetin. Subsequently, **22** was esterified with substituted benzoic acids using EDC and DMAP to afford **23**. The cyclization reaction was then carried out under basic conditions using TBAB as a catalyst, followed by deprotection with THP to yield the flavonol skeleton. Finally, the 4-bromo functionality was reduced using LiAlH_4_ to afford polyhydroxy flavonols **24** (Scheme 6). Notably, this approach streamlines the three-step sequence by integrating the rearrangement and cyclization into a single operation, thereby improving step economy and reducing reaction time. Direct cyclization proceeds under mild conditions using K_2_CO_3_/TBAB in toluene at 90 °C, avoiding strong acids or Lewis acids required in conventional protocols. Final deprotection proceeds without compromising the flavonol core, underscoring the practicality of this method.

#### 2.3.2. Synthesis of Polyhydroxy Flavonols **28**

**Scheme 7:**
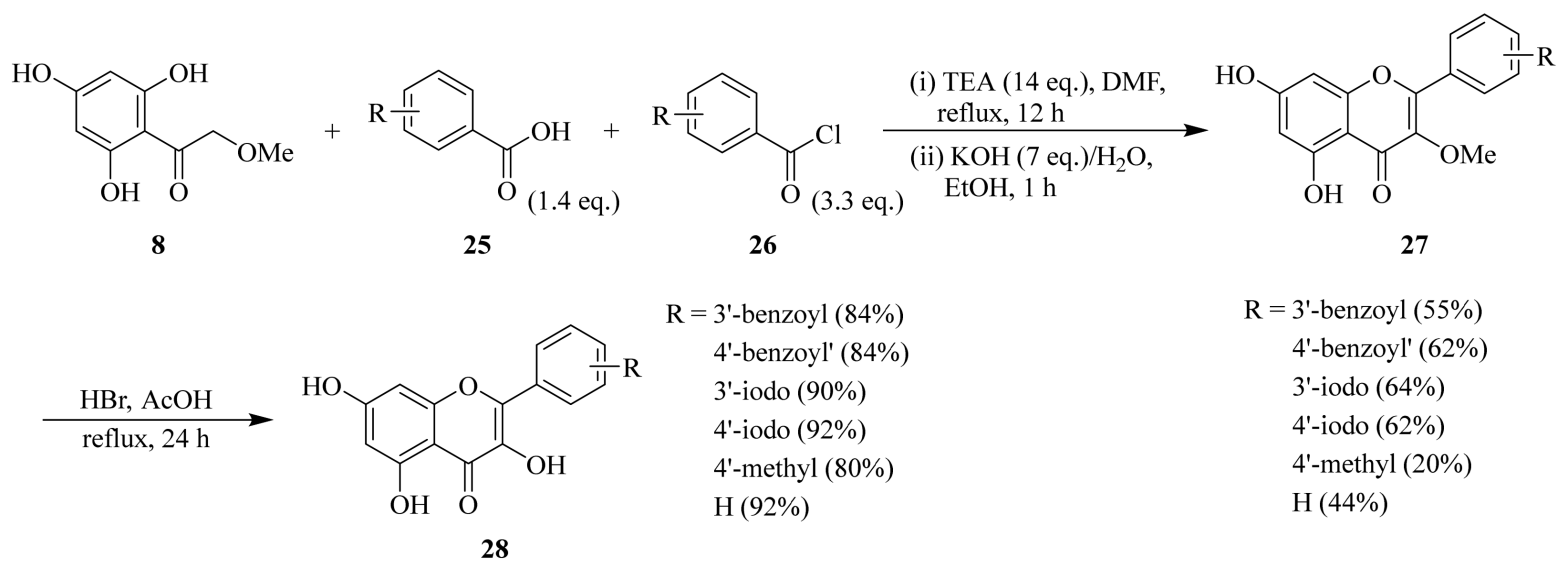
One-pot synthetic route of polyhydroxy flavonols **28**.

Despite these advances, stepwise approaches involving protection, acylation, cyclization, and deprotection suffer from lengthy procedures and moderate yields. To address these limitations, streamlined synthetic strategies have been developed. In 2014, Forbes et al. [47] reported a modified one-pot Baker–Venkataraman rearrangement for the synthesis of flavonols, adapting the method originally developed by Ichikawa et al. [48]. Methoxy phloroacetophenone **8** was treated with substituted benzoic acid **25** and the corresponding benzoyl chloride **26** in the presence of triethylamine and DMF. This mixed anhydride strategy facilitated acylation and in situ cyclization under basic conditions, directly affording the flavonol methyl ether intermediate without isolating the triester intermediate. Demethylation to yield polyhydroxy flavonol **28** was initially attempted using BBr_3_/CHCl_3_ or Na_2_S/DMF, but both proved unsatisfactory: BBr_3_ afforded only partial demethylation due to its water sensitivity; Na_2_S failed because deprotonation of the phenolic hydroxyl groups rendered the aromatic ring electron-rich and the methoxy groups unreactive. Complete demethylation was ultimately achieved using HBr/AcOH (Scheme 7). This protocol offers advantages over traditional stepwise approaches, including elimination of intermediate isolation, reduced solvent consumption, simplified workup, shorter reaction time, and improved yields, making it suitable for scalable synthesis of diverse flavonol analogs.

#### 2.3.3. Synthesis of Houttuynoid B **35**

**Scheme 8:**
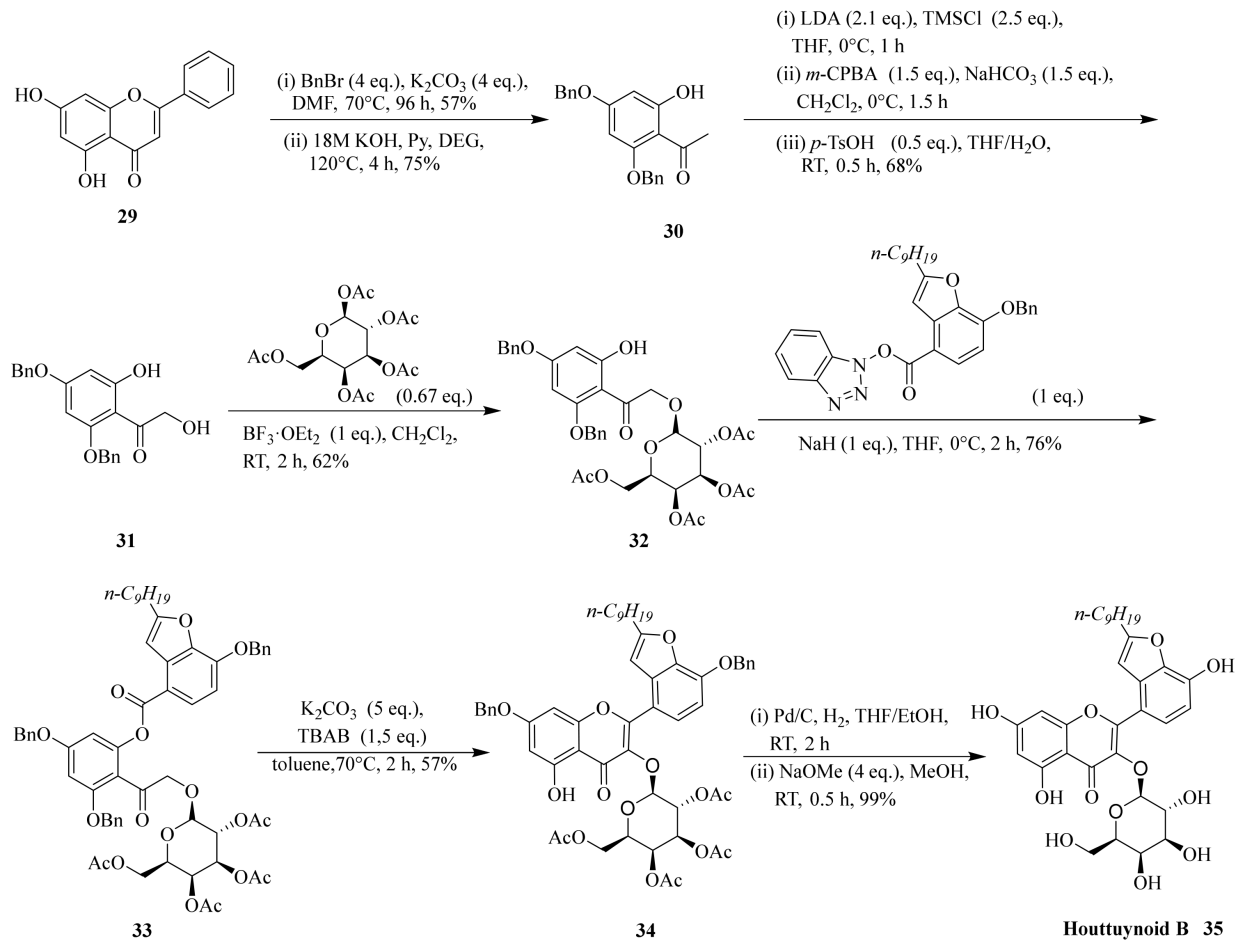
Synthetic route of Houttuynoid B **35**.

In 2016, Kerl et al. [49] reported the first total synthesis of Houttuynoid B **35**, employing a Baker–Venkataraman rearrangement on a pre-glycosylated substrate to construct the flavonoid skeleton. The synthesis started from chrysin **29**, which was converted to **31** via bis o-benzylation, retro-aldol reaction, and Rubottom α-oxidation. Subsequent glycosylation with peracetylated *β*-galactose afforded **32**, which was coupled with the HOBT-activated benzofuran derivative to give aryl ester **33**. Using potassium carbonate as a base in the presence of TBAB as a phase-transfer catalyst, ester **33** underwent the desired Baker–Venkataraman rearrangement with concomitant in situ cyclization of the intermediate 1,3-diketone to afford **34**. Finally, hydrogenolysis followed by deacetylation yielded Houttuynoid B **35** (Scheme 8). Notably, this work achieved the first total synthesis of a glycosylated flavonoid via a pre-glycosylated Baker–Venkataraman rearrangement, a transformation previously considered challenging due to the instability of glycosidic bonds under basic conditions. The mixed Bn/Ac protection strategy enabled orthogonal deprotection. However, the nine-step route (11% overall yield) features a modest 57% yield in the key rearrangement step and requires sequential deprotection, indicating room for further optimization.

#### 2.3.4. Synthesis of (±)-Sanggenol F **51**

**Scheme 9:**
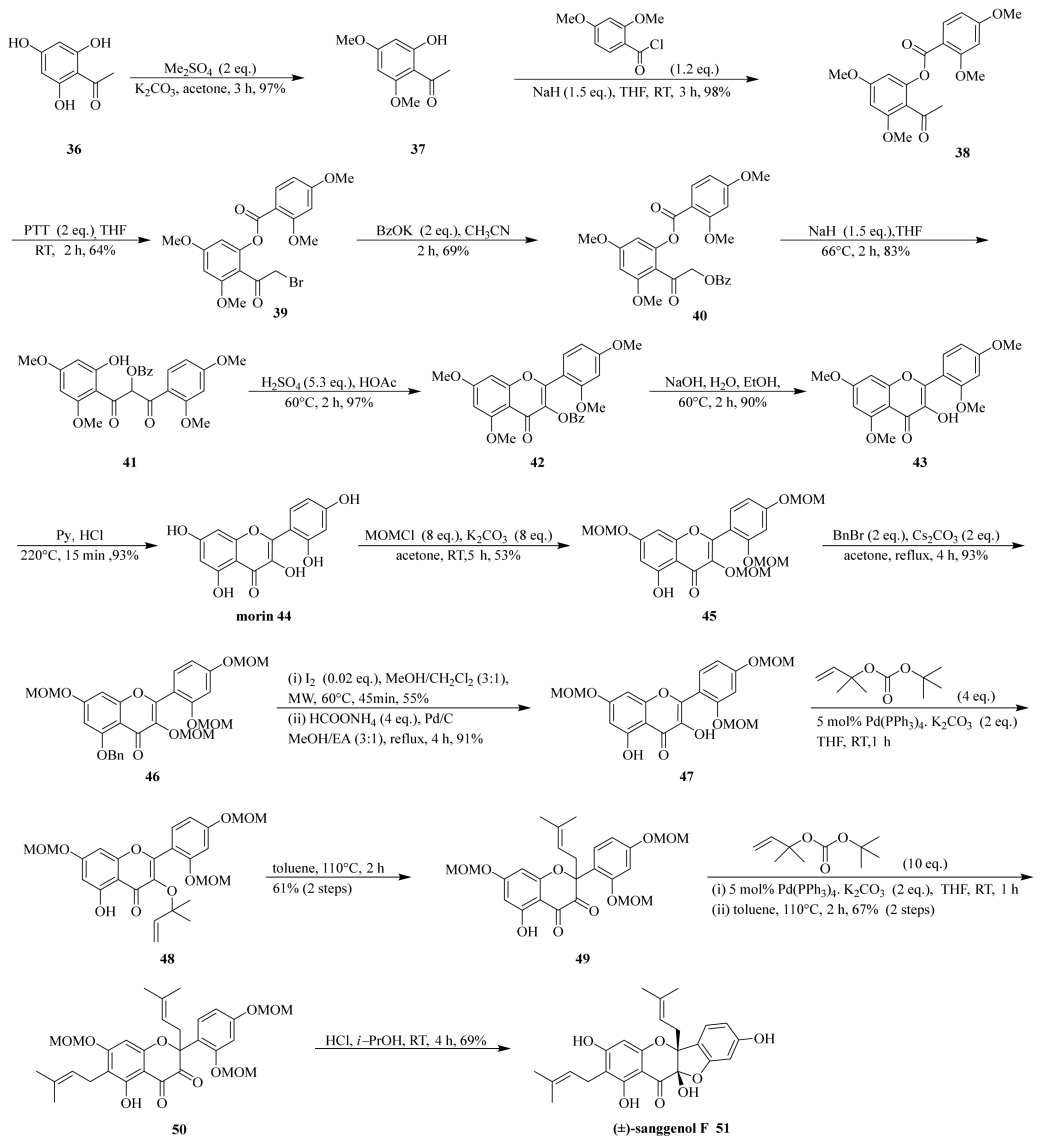
Synthetic route of (±)-sanggenol F **51**.

In 2017, Sheng et al. [50] reported the total synthesis of (±)-sanggenol F **51**. The synthesis commenced from commercially available 2,4,6-trihydroxyacetophenone **36**, which was methylated with Me_2_SO_4_ and K_2_CO_3_ in refluxing acetone to afford **37**. Subsequent esterification with 2,4-dimethoxybenzoyl chloride in the presence of NaH in THF provided ester **38**. α-Bromination of **38** with PTT was carried out with batchwise addition of the reagent to prevent hydrolysis of the ester **39**. Nucleophilic substitution with potassium benzoate in refluxing acetonitrile afforded **40**. The Baker–Venkataraman rearrangement of **40** with NaH in refluxing THF proceeded to afford **41**. Cyclization under acidic conditions gave **42**, followed by saponification to remove the benzoate group, affording **43**. Finally, complete demethylation with pyridine hydrochloride at 220 °C yielded morin **44**, which was then elaborated to sanggenol F **51** through sequential prenylation and Claisen rearrangement (Scheme 9). This route to morin **44** features the Baker–Venkataraman rearrangement to construct the flavonoid skeleton without strong oxidants, and the final demethylation delivers the natural product in high yield. However, the overall sequence is relatively lengthy, and the harsh conditions required for the demethylation may limit its scalability.

#### 2.3.5. Synthesis of 4′-Substituted Kaempferols **59**

**Scheme 10:**
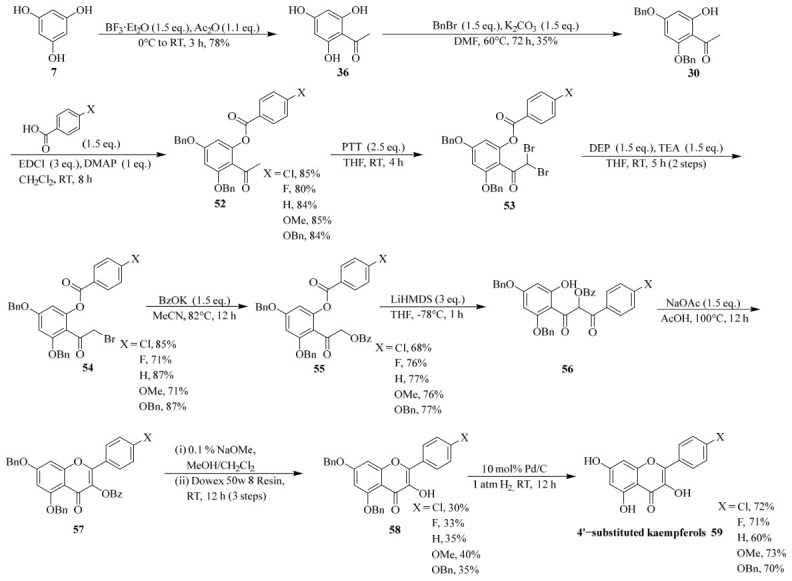
Synthetic route of 4′-substituted kaempferols **59**.

In 2020, Kim et al. [51] reported the synthesis of 4′-substituted kaempferols **59** via the Baker–Venkataraman rearrangement. The synthesis commenced with Lewis-acid-catalyzed acylation of phloroglucinol **7** to afford 2,4,6-trihydroxyacetophenone **36**, which was subsequently bis-benzylated to provide **30**. Diversification at the 4′-position was introduced via EDCI-mediated coupling of **30** with various substituted benzoic acids to form phenolic esters **52**. The resulting methyl ketones **52** were subjected to α-bromination, most efficiently achieved via a bis-bromination-selective reduction sequence to give the mono-bromide intermediates **54**, followed by nucleophilic displacement with potassium benzoate to afford benzoates **55**. Subsequent treatment with LiHMDS promoted the Baker–Venkataraman rearrangement to generate 1,3-diketone intermediate **56**. Then **56** was subjected to dehydrative cyclization under acidic conditions with heating to afford the perbenzylated kaempferol intermediate **57**. Finally, hydrolysis of the benzoate ester **57** furnished perbenzylated kaempferol **58**, which underwent global deprotection via hydrogenolysis over Pd/C to afford the target flavonol, 4′-substituted kaempferols **59** (Scheme 10). This route features benzyl protecting groups to shield the C5 and C7 hydroxyl groups, preventing side reactions, and the Baker–Venkataraman rearrangement constructs the flavonol skeleton under mild conditions with LiHMDS. However, the overall sequence is relatively lengthy, and the moderate yield of the cyclization step may limit overall synthetic efficiency.

#### 2.3.6. Synthesis of Cycloicaritin **71**

**Scheme 11:**
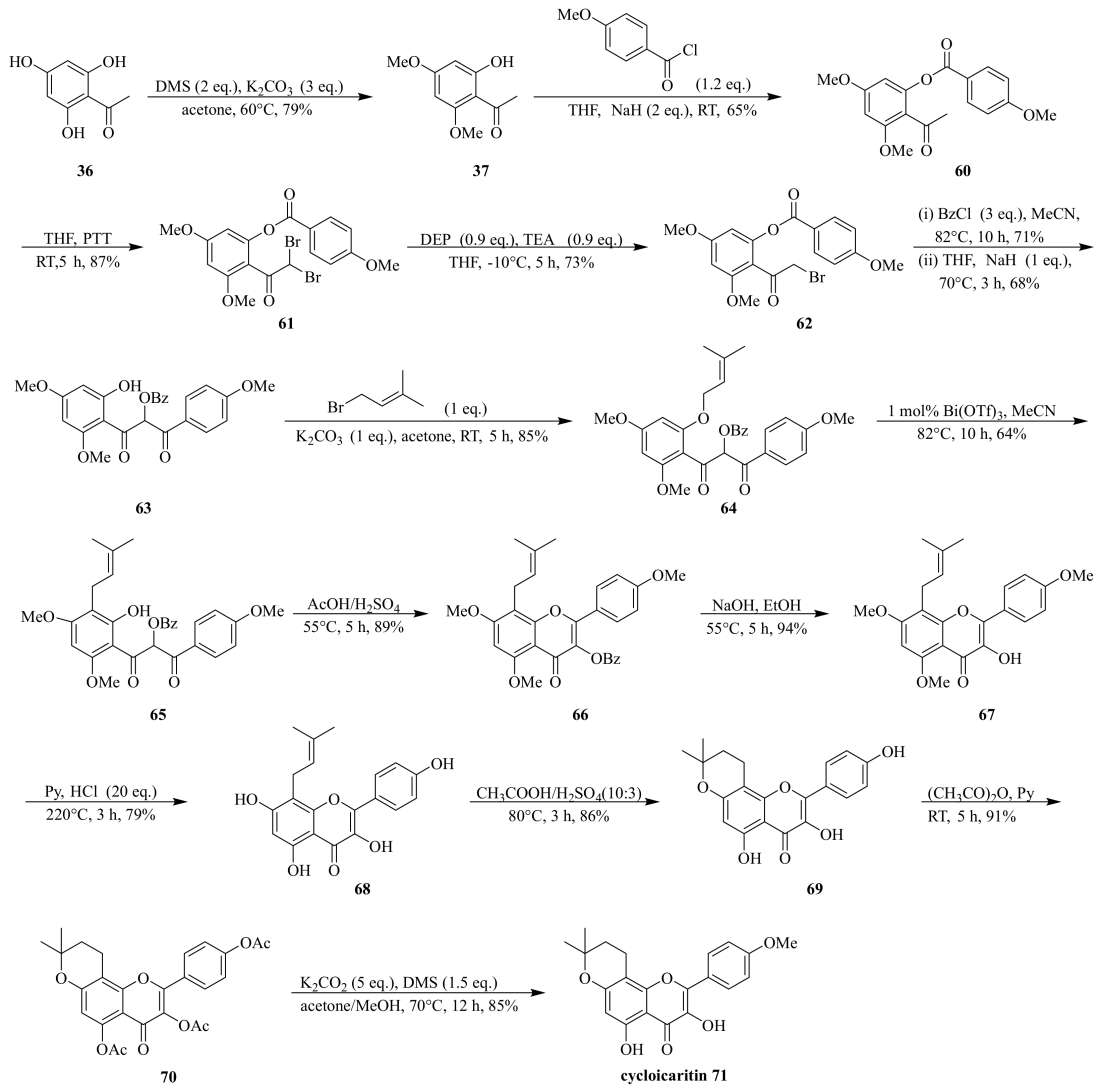
Synthetic route of cycloicaritin **71**.

In 2019, Liu et al. [52] reported a total synthesis of cycloicaritin **71**. The synthesis commenced with 2,4,6-trihydroxyacetophenone **36**. Methylation with DMS afforded **37**. Esterification of **37** with 4-methoxybenzoyl chloride gave the phenolic ester **60**. α-Bromination of **60** using PTT afforded dibromide **61**. Selective debromination with DEP and TEA gave monobromide **62**. Treatment of **62** with benzoyl chloride gave the corresponding benzoate, which underwent Baker–Venkataraman rearrangement with NaH to afford 1,3-diketone **63**. Prenylation of **63** with prenyl bromide gave **64**, which underwent ortho-Claisen rearrangement with Bi(OTf)_3_ to afford **65**. Subsequent cyclization under acidic conditions furnished **66**. Removal of the benzoyl group under basic conditions gave **67**, followed by demethylation with pyridine hydrochloride to afford polyhydroxy flavonol **68**. Finally, cyclization of the prenyl group in **68** under acidic conditions gave the pyran ring **69**, followed by acetylation, methylation at the 4′-position, and deacetylation to furnish cycloicaritin **71** (Scheme 11). This route features pre-flavonoid prenylation coupled with inexpensive Bi(OTf)**_3_**-catalyzed ortho-rearrangement, circumventing the microwave irradiation or costly europium catalysts required in prior approaches. However, the lengthy linear sequence with multiple protection and deprotection cycles poses scalability challenges for industrial application.

#### 2.3.7. Synthesis of Icaritin **77**

**Scheme 12:**
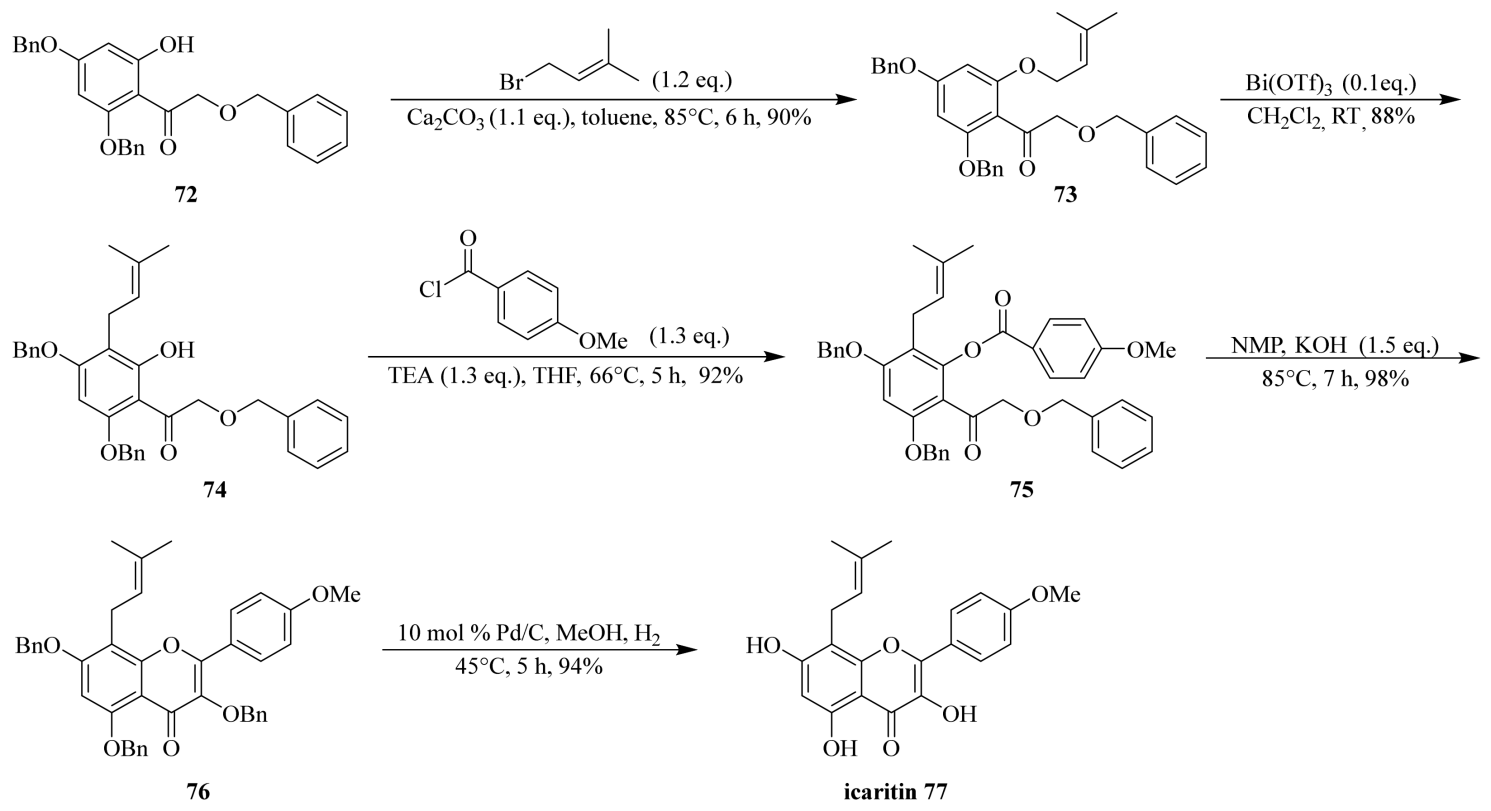
Improved synthetic route of icaritin **77** via Baker–Venkataraman rearrangement.

In 2022, Sui et al. [53] reported a novel synthetic route to icaritin **77**. The synthesis commenced with compound **72**. Prenylation with prenyl bromide gave **73**, which underwent Bi(OTf)_3_-catalyzed ortho-Claisen rearrangement to afford **74**. Esterification of **74** with 4-methoxybenzoyl chloride gave **75**, followed by Baker–Venkataraman rearrangement under basic conditions to furnish **76**. Finally, hydrogenolysis over Pd/C removed the benzyl-protecting groups to afford icaritin **77** in five steps (Scheme 12). This route features benzyl protection for facile one-pot hydrogenolytic removal and pre-flavonoid prenylation to suppress rearrangement by-products. This contrasts with the route by Liu et al. [52] (Scheme 11), which suffers from excessive methoxy protection and deprotection cycles and harsh demethylation conditions. The strategic use of benzyl groups enables milder reaction conditions and superior scalability, overcoming the industrial limitations of prior approaches.

The Baker–Venkataraman rearrangement offers an efficient route to flavonols from readily accessible starting materials under mild conditions, offering operational simplicity and broad substrate scope. However, the conventional protocol is limited by multi-step operations and moderate yields. Optimization, including improved catalytic systems, one-pot strategies, and streamlined protecting-group approaches, holds potential to enhance the overall efficiency of the rearrangement. With these refinements, the Baker–Venkataraman rearrangement is poised to become an increasingly valuable tool for flavonol synthesis.

### 2.4. Algar–Flynn–Oyamada (AFO) Reaction

**Scheme 13:**
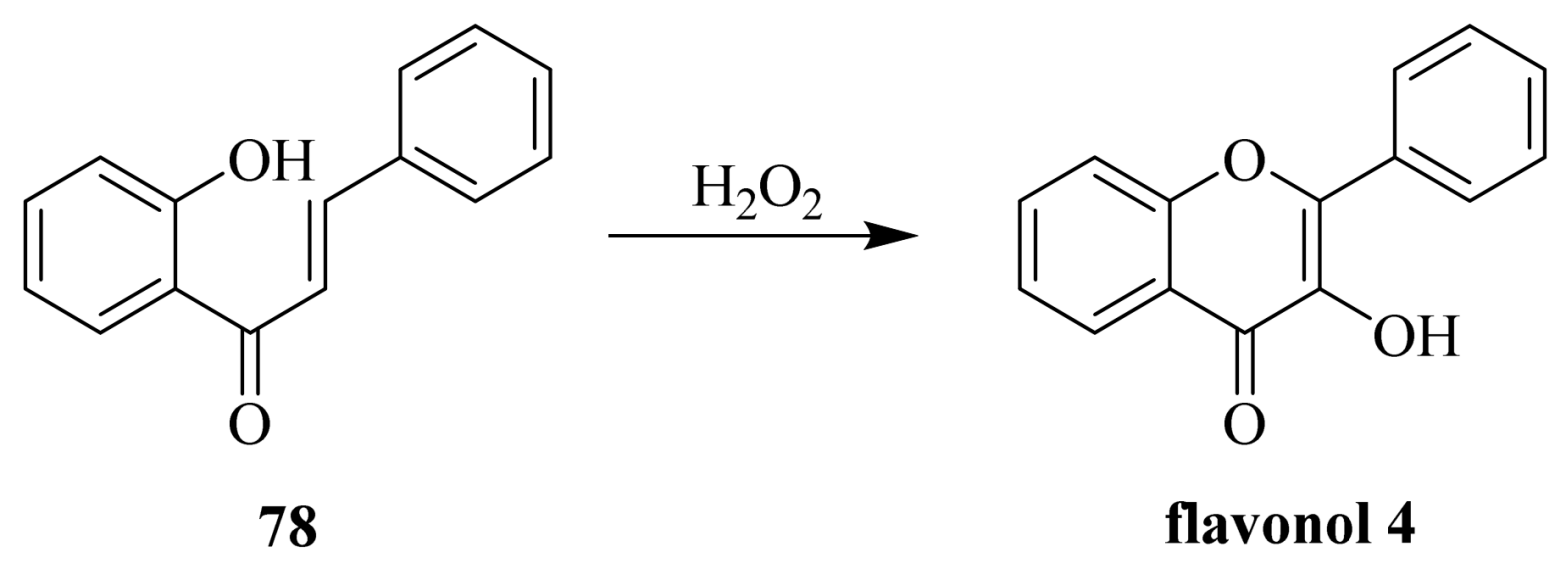
Synthetic route of flavonol **4** via the Algar–Flynn–Oyamada reaction.

The Algar–Flynn–Oyamada (AFO) reaction was independently reported by Algar, Flynn, and Oyamada in 1934 [54]. It proceeds under mild basic conditions through the oxidative cyclization of 2′-hydroxychalcone **78** with H_2_O_2_, affording flavonol **4** (Scheme 13). Owing to its operational simplicity, avoidance of toxic reagents, high atom economy, and environmentally benign profile, the AFO reaction has been widely adopted for flavonol synthesis.

**Scheme 14:**
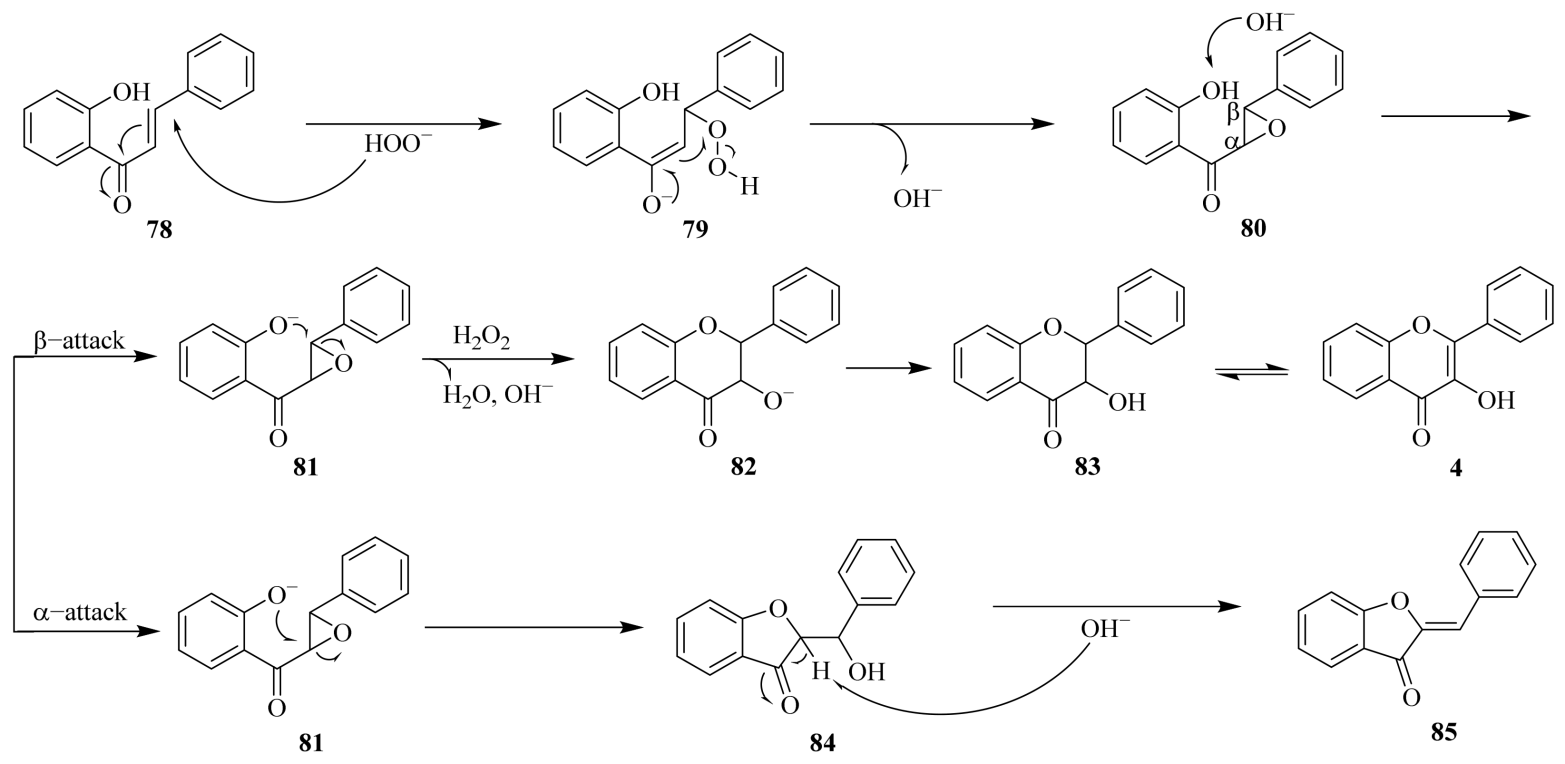
The AFO reaction mechanism.

The mechanism of the AFO reaction has been the subject of considerable recent investigation. Under alkaline conditions, 2′-hydroxychalcone **78** is proposed to undergo oxidation by H_2_O_2_ to form the epoxide intermediate **80**. If the phenoxide anion within the epoxide attacks the carbonyl β-position, dihydroflavonol **83** is generated, which is subsequently oxidized to flavonol **4**. Conversely, if the carbonyl α-position is targeted, the five-membered cyclic aurone by-product **85** is produced (Scheme 14) [55,56].

The AFO reaction is widely employed for flavonol synthesis, yet the involvement of epoxide intermediates in its mechanism continues to be debated. Although some evidence supports epoxide formation under certain conditions, particularly for 6′-substituted chalcones [57,58], no direct characterization of such species has been unequivocally verified [59]. Based on DFT calculations, Dean and Podimuang [60] proposed an alternative direct oxidative cyclization route that bypasses epoxide intermediates for unsubstituted substrates. Experimental studies further suggest that this mechanism is both kinetically and thermodynamically more favorable for unsubstituted substrates.

#### 2.4.1. Synthesis of Quercetagetin **90**

**Scheme 15:**
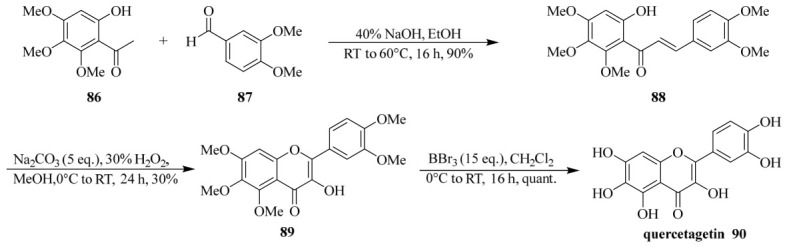
Synthetic route of quercetagetin **90**.

In the AFO reaction, quantities of homogeneous base catalysts such as NaOH, KOH, or pyrrolidine are typically employed. However, chalcone substrates are sensitive to strongly basic environments. In 2021, Bulut et al. [61] described a streamlined and efficient synthesis of quercetagetin **90** using modified AFO conditions. The synthesis began with the aldol condensation of 3,4,5-trimethoxyacetophenone **86** and 3,4-dimethoxybenzaldehyde **87** in the presence of NaOH to afford 2′-hydroxychalcones **88**. Subsequently, the AFO reaction was successfully carried out using H_2_O_2_ as the oxidant and Na_2_CO_3_ as the base to achieve the oxidative cyclization of **88** to flavonol **89**. Finally, demethylation with BBr_3_ provided the target product, quercetagetin **90** (Scheme 15). In this improved protocol, the conventional strong base NaOH was replaced with Na_2_CO_3_, significantly optimizing the reaction process. Compared to NaOH, Na_2_CO_3_ provides milder conditions, and exhibits improved stability across a range of solvents. These modifications help suppress side reactions commonly promoted by strong alkalis, thereby improving the overall synthetic yield.

#### 2.4.2. One-Pot Synthesis of Polyhydroxy Flavonols

**Scheme 16:**
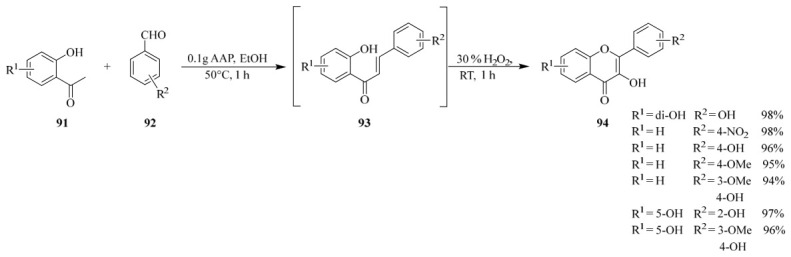
One-pot synthetic route of flavonols **94**.

To reduce by-product formation in the AFO reaction and overcome limitations of conventional basic media, Tamaddon et al. [62] developed a bio-based catalyst, alkaline amylopectin (AAp). The catalyst has a high base capacity of 7.3 mmol HO^−^/g and exhibits significant catalytic activity in both aldol condensation and AFO reactions. The synthetic sequence involves aldol condensation between 2′-hydroxyacetophenone **91** and aromatic aldehydes **92** to afford 2′-hydroxychalcones **93**, followed by oxidative cyclization with H_2_O_2_ in the presence of AAp to furnish various substituted flavonols **94** in 94–98% yield (Scheme 16). Compared with earlier AFO protocols, this one-pot procedure achieves excellent yields without aurone formation using low catalyst loading. The key innovation is the use of recoverable AAp as a bio-based heterogeneous catalyst, which can be recycled five times. However, the catalyst preparation is relatively complex, and the protocol still requires H_2_O_2_ as the oxidant.

**Scheme 17:**
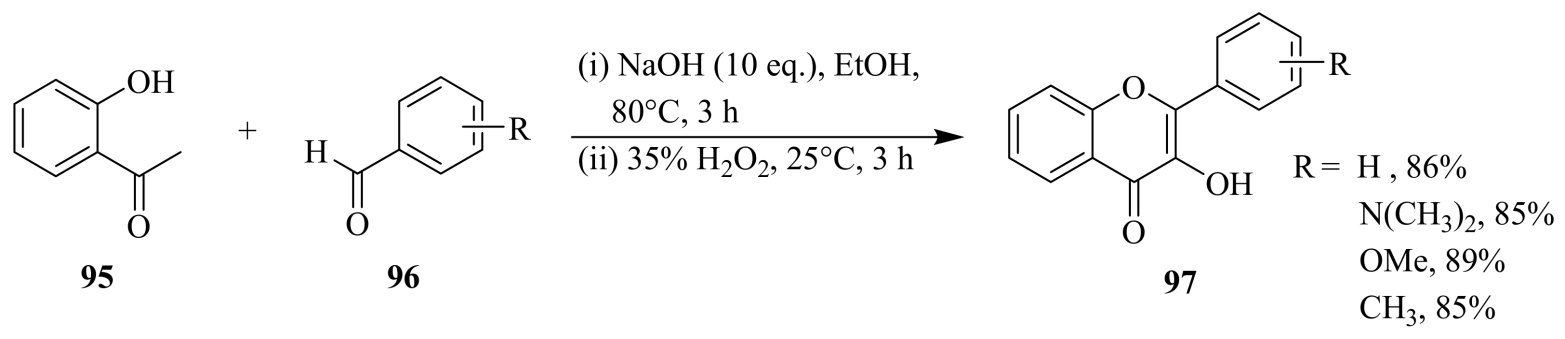
One-pot synthetic route of flavonols **97**.

The AFO reaction involves three distinct steps, condensation to form the chalcone, cyclization to generate the dihydroflavonol, and oxidation to yield the flavonol. Yan et al. [63] analyzed the reaction conditions and identified commonalities, enabling the development of an optimized one-pot AFO procedure. 2′-hydroxyacetophenone **95** and substituted benzaldehyde **96** were combined in ethanol containing NaOH and refluxed at 80 °C for 3 h. After cooling to room temperature, 35% H_2_O_2_ was added, and the mixture was stirred for an additional 3 h. This afforded substituted flavonols **97** (Scheme 17). Compared with traditional stepwise conditions, this one-pot protocol achieved higher yields. Response surface methodology optimized base loading, solvent volume, and water content. However, the protocol requires a large excess of NaOH and a relatively complex optimization process, which may limit its utility for rapid reaction development.

**Scheme 18:**
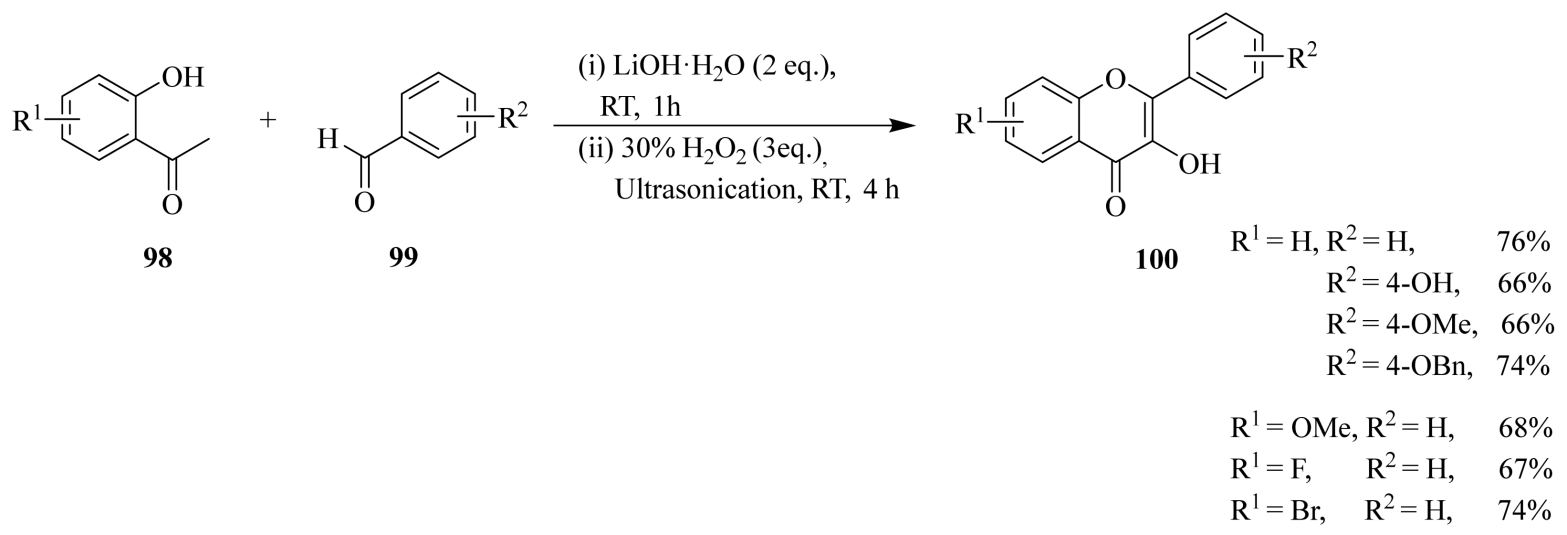
One-pot synthetic route of flavonols **100**.

Aligned with green chemistry principles, Kumar et al. [64] developed a one-pot method for flavonol synthesis by optimizing the traditional AFO reaction. The method utilizes an “on-water” strategy, a contemporary approach that minimizes waste while affording an environmentally benign reaction medium. The process is further enhanced by sonochemistry, which improves reaction homogeneity, kinetics, and yield through ultrasound activation. Starting from the substituted 2′-hydroxyacetophenone **98** and the corresponding substituted benzaldehyde **99**, this reaction employs water as the sustainable solvent, with LiOH·H_2_O as the mild base and H_2_O_2_ as the oxidant. This design enables direct one-pot conversion to flavonols without isolating the intermediate chalcone, affording flavonols **100** in 62–76% yield (Scheme 18). This route achieves moderate to good yields with operational simplicity and low cost, using water as the sole solvent and ultrasound to enhance kinetics. The key innovation lies in combining on-water conditions with sonochemistry, eliminating the need for intermediate isolation and external heating. However, the yields are modest compared to some other one-pot AFO protocols, and ultrasound reliance may limit scalability.

**Scheme 19:**
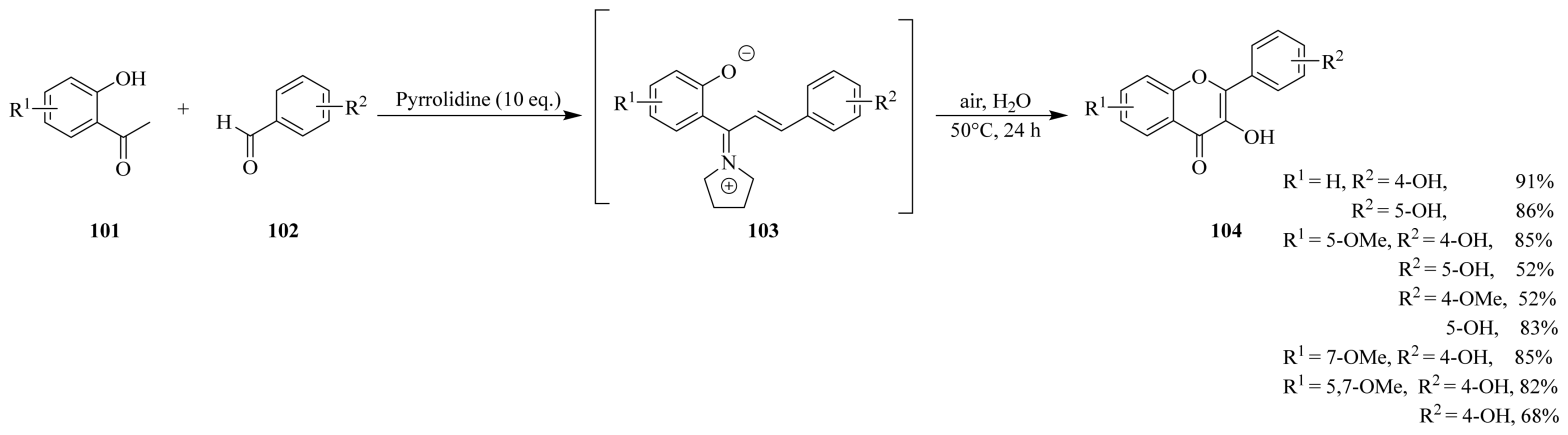
One-pot synthetic route of flavonols **104**.

The conversion of 2′-hydroxyacetophenone and benzaldehyde to flavonols via the AFO reaction requires both alkaline conditions and an oxidant. Expanding on the AFO transformation, Xiong et al. [65] developed an innovative catalytic approach using pyrrolidine. Under aerobic conditions in water, pyrrolidine acts as an organocatalytic base to promote the direct, one-pot synthesis of flavonols **104** from 2′-hydroxyacetophenones **101** and benzaldehydes **102** (Scheme 19). This route simplifies the synthetic sequence, employs water as a green solvent, and tolerates diverse substrates, highlighting its promise for practical application.

#### 2.4.3. Synthesis of Icaritin **77**

**Scheme 20:**
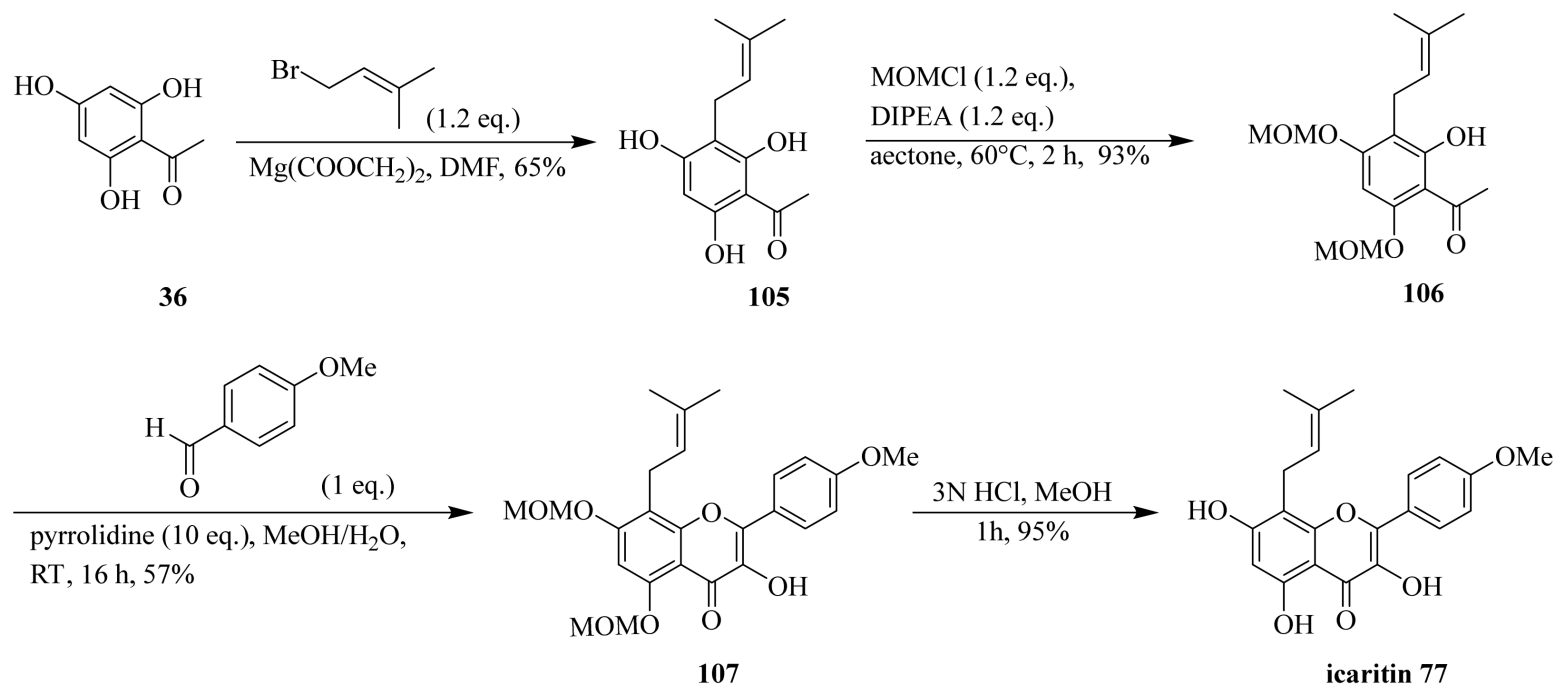
Synthetic route of icaritin **77** via AFO reaction.

In 2022, Zhang et al. [66] developed a four-step total synthesis of icaritin **77** from 2,4,6-trihydroxyacetophenone **36**. Magnesium succinate-promoted C-prenylation of **36** afforded **105**, which upon selective MOM protection gave **106**. Attempted flavonol construction under classical AFO conditions using NaOH/H_2_O_2_ failed due to prenyl group incompatibility. To overcome this, a pyrrolidine/air oxidation system was employed as a mild alternative, enabling **106** to condense with 4-methoxybenzaldehyde in methanol/water (1:3) to furnish **107**. Final acid deprotection provided icaritin **77** in 33% overall yield (Scheme 20). This approach resolves both regioselectivity and functional group compatibility challenges. However, the moderate yield and solvent sensitivity in the flavonol construction step remain as limitations, warranting further optimization of oxidative cyclization conditions.

#### 2.4.4. Synthesis of Morin **44**

**Scheme 21:**
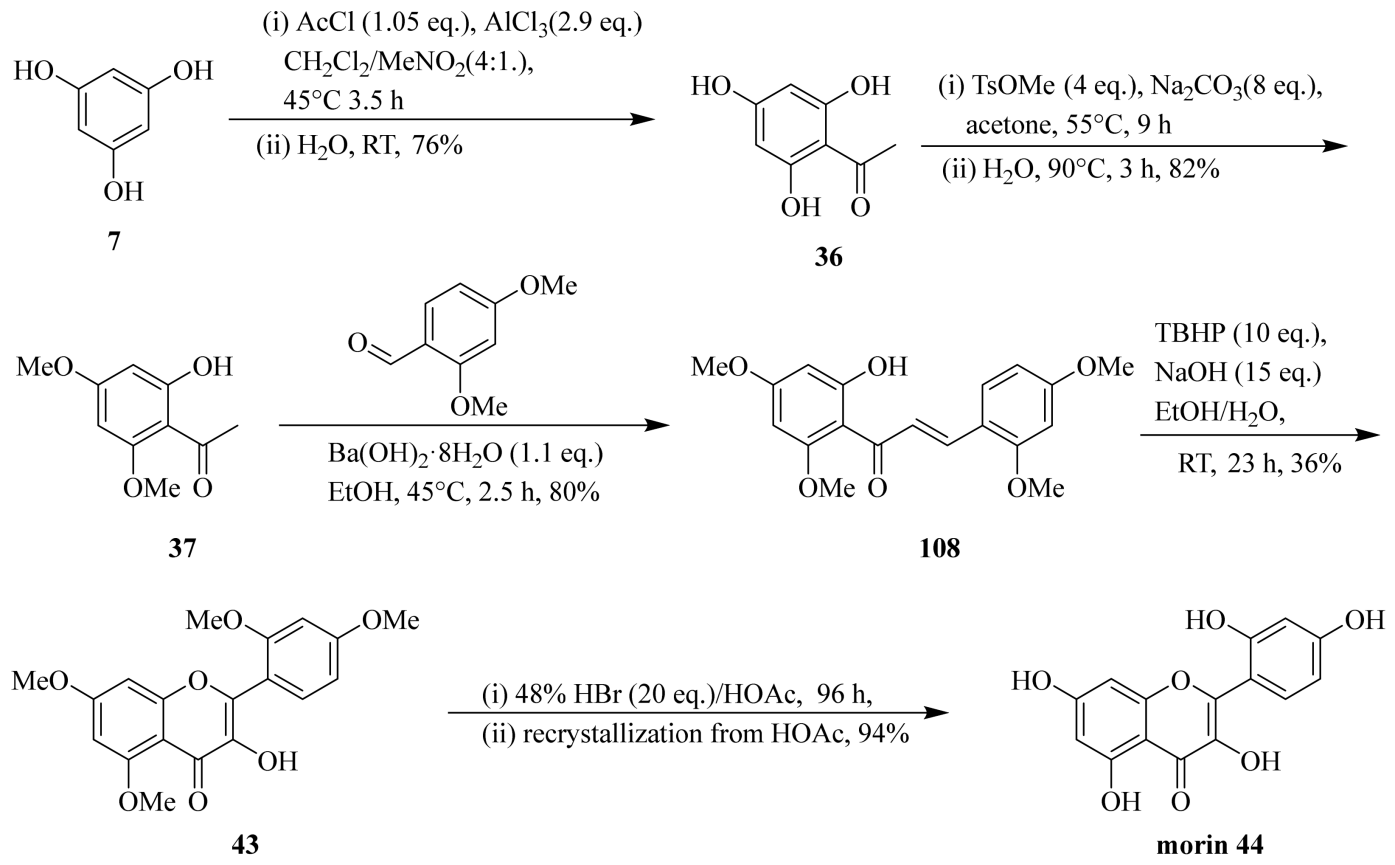
Synthetic route of morin **44**.

In 2023, Gurzadyan et al. [67] reported an improved AFO reaction as part of a streamlined five-step synthesis of morin **44**. The synthesis commenced with Friedel–Crafts acylation of phloroglucinol **7**, affording **36**. The hydroxyl groups were then protected by methylation using TsOMe/Na_2_CO_3_ to give **37**. Subsequent treatment of **37** with 2,4-dimethoxybenzaldehyde in the presence of Ba(OH)_2_·8H_2_O afforded chalcone **108**. Oxidative cyclization of **108** with TBHP/NaOH under AFO conditions then furnished flavonol **43**. Finally, demethylation with 48% HBr in acetic acid yielded morin **44** (Scheme 21). This route offers practical improvements through five linear steps with chromatography-free purification and scalability to multigram-scale production, though the modest overall yield of 18.3% reflects significant by-product formation in the oxidative cyclization step. Addressing these competing pathways represents a key challenge for enhancing the overall efficiency and generality of this scalable approach.

#### 2.4.5. Synthesis of Fisetin **114**

**Scheme 22:**
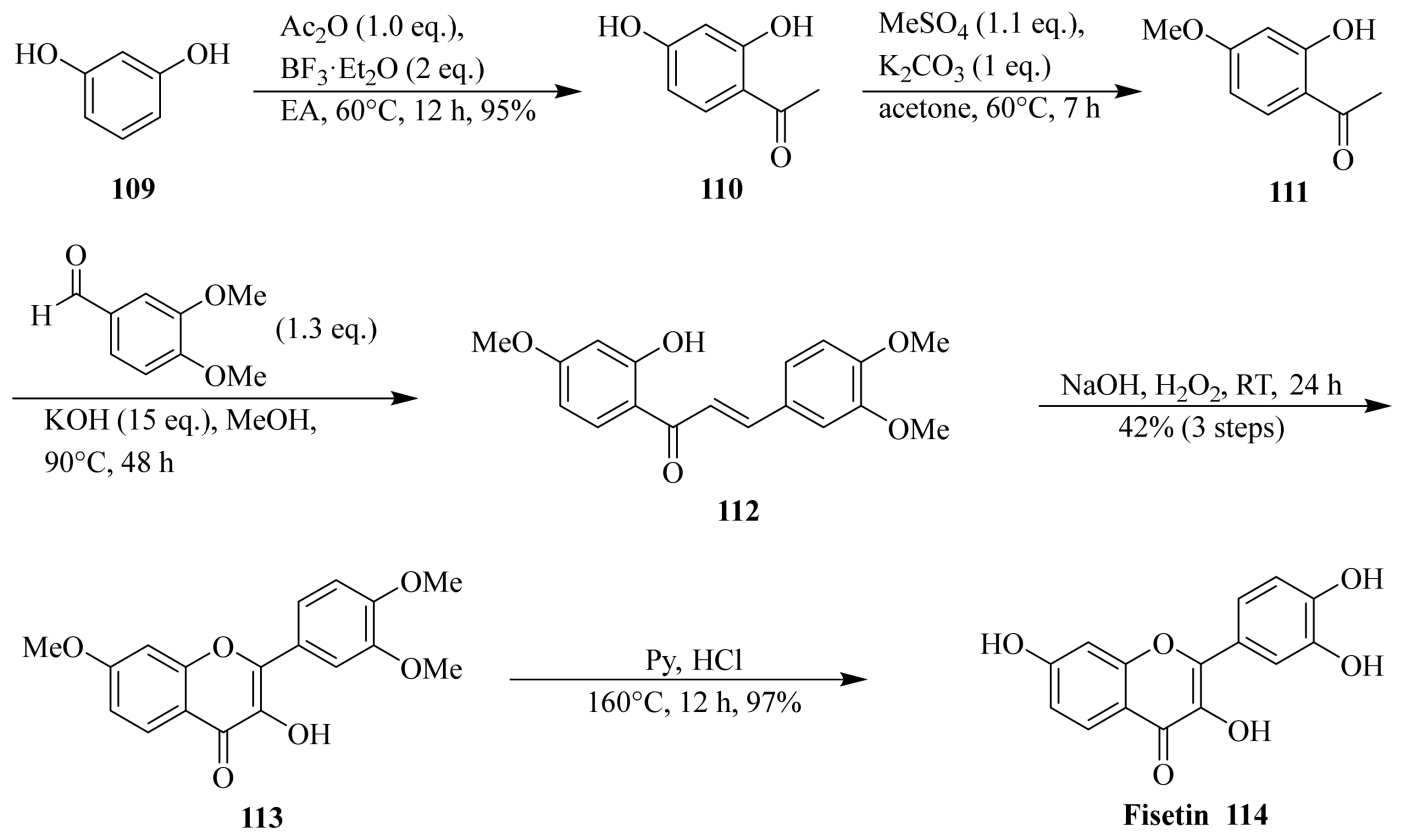
Synthetic route of fisetin **114**.

Fisetin, a polyhydroxy flavonol, has been widely studied as a potential therapeutic agent for multiple diseases. In 2021, Chu et al. [68] described a concise total synthesis of fisetin **114**. The route began with Friedel–Crafts acylation of resorcinol **109** to give 2,4-dihydroxyacetophenone **110**, which was methylated to afford **111**, improving its stability under basic conditions. Condensation of **111** with benzaldehyde yielded 2′-hydroxychalcone **112**. Initial attempts at the AFO reaction using 0.5 M NaOH and 35% H_2_O_2_ in water were unsuccessful due to the poor solubility of the methylated chalcone in the aqueous base. Switching to an ethanolic solution of NaOH and H_2_O_2_ afforded the flavonol product in only 20% yield. Further optimization revealed that gradual, simultaneous addition of aqueous NaOH and H_2_O_2_ enabled efficient formation of **113**. Finally, demethylation with pyridine hydrochloride delivered fisetin **114** (Scheme 22). This five-step route achieves approximately 40% overall yield with chromatography-free purification, though the AFO oxidation requires precise control of reagent addition to avoid yields as low as 20% under unoptimized conditions. The key innovation lies in the continuous, simultaneous addition strategy that overcomes the poor aqueous solubility of methylated chalcones, a critical challenge for scalable flavonol synthesis.

The AFO reaction offers several practical advantages, including mild conditions, broad substrate scope, and operational simplicity. Recent advances have focused on green chemistry principles, with the development of bio-based catalysts, on-water strategies, ultrasound assistance, and chromatography-free purification. These improvements have significantly enhanced the efficiency, selectivity, and scalability of the AFO transformation. However, challenges remain, including aurone by-products and moderate yields for certain substituted substrates. Future optimization of catalytic systems and reaction conditions will further broaden the applicability of this reliable strategy for flavonol synthesis.

### 2.5. DMDO Oxidative Reaction

**Scheme 23:**
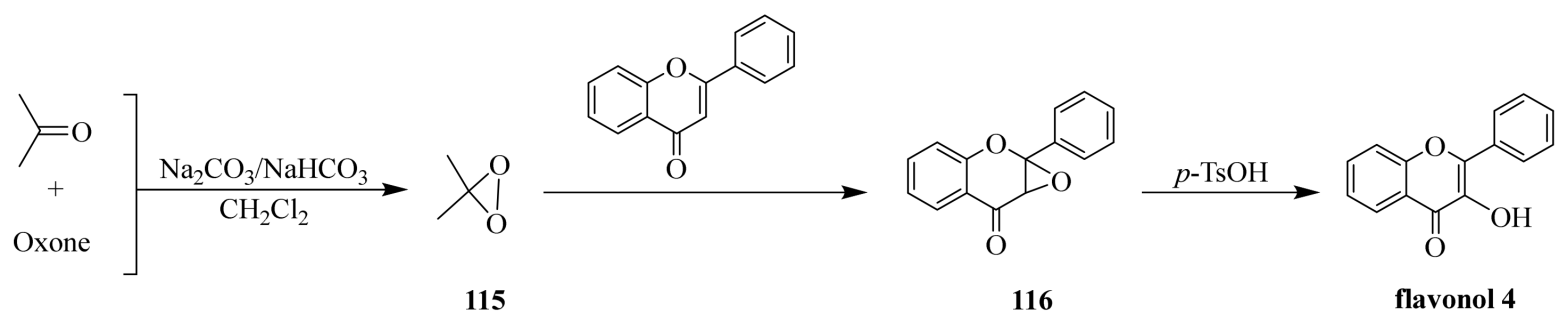
Synthetic route of flavonol **4** via the DMDO oxidative reaction.

DMDO is a mild and versatile oxidizing agent widely used for the stereoselective epoxidation of double bonds. The oxidation proceeds through a sequence in which oxone oxidizes acetone to form peroxyacetone **115**. At room temperature, DMDO reacts with the double bond via electrophilic addition to generate epoxide **116**. Subsequent treatment with TsOH opens the epoxide ring to afford the enol form, flavonol **4** (Scheme 23). This method is characterized by high stereoselectivity, minimal by-product formation, and operational simplicity [69,70].

#### 2.5.1. Synthesis of Kaempferol 3-O-Neohesperidoside **123**

**Scheme 24:**
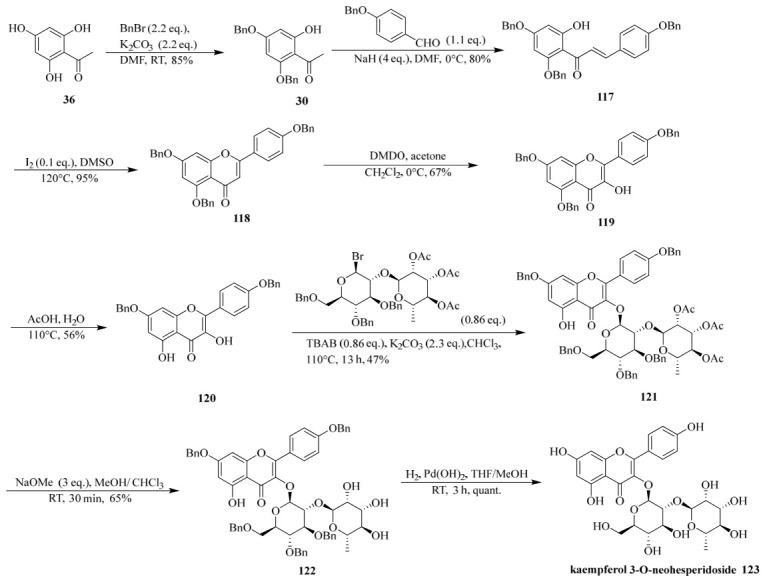
Synthetic route of kaempferol 3-O-neohesperidoside **123**.

In 2010, Yamasaki et al. [71] reported the synthesis of kaempferol 3-O-neohesperidoside **123** from 2,4,6-trihydroxyacetophenone **36**. The synthesis commenced with selective benzylation to afford **30**, followed by aldol condensation with 4-(benzyloxy)benzaldehyde under basic conditions to give chalcone **117**. Oxidative cyclization of **117** using I_2_/DMSO at 120 °C furnished flavone **118**. Oxidation with DMDO selectively introduced a hydroxyl group at the C3 position to yield flavonol **119**. Due to intramolecular hydrogen bonding between the 3-OH and the carbonyl group that interfered with glycosylation, the 5-O-benzyl group was removed by heating in aqueous acetic acid to give **120**. Finally, glycosylation of **120** with neohesperidosyl bromide under phase-transfer conditions, then sequential removal of the acetyl and benzyl protecting groups, furnished the target kaempferol 3-O-neohesperidoside **123** (Scheme 24). This route represents the first total synthesis of kaempferol 3-O-neohesperidoside **123**. It introduces DMDO as a mild reagent for selective C3 hydroxylation of flavones under neutral, metal-free conditions, while the I_2_/DMSO cyclization results in high yield. However, the linear sequence suffers from a very low overall yield of 7% due to multiple protection/deprotection steps and is not readily adaptable to other flavonoid scaffolds.

#### 2.5.2. Synthesis of Icaritin **77**

**Scheme 25:**
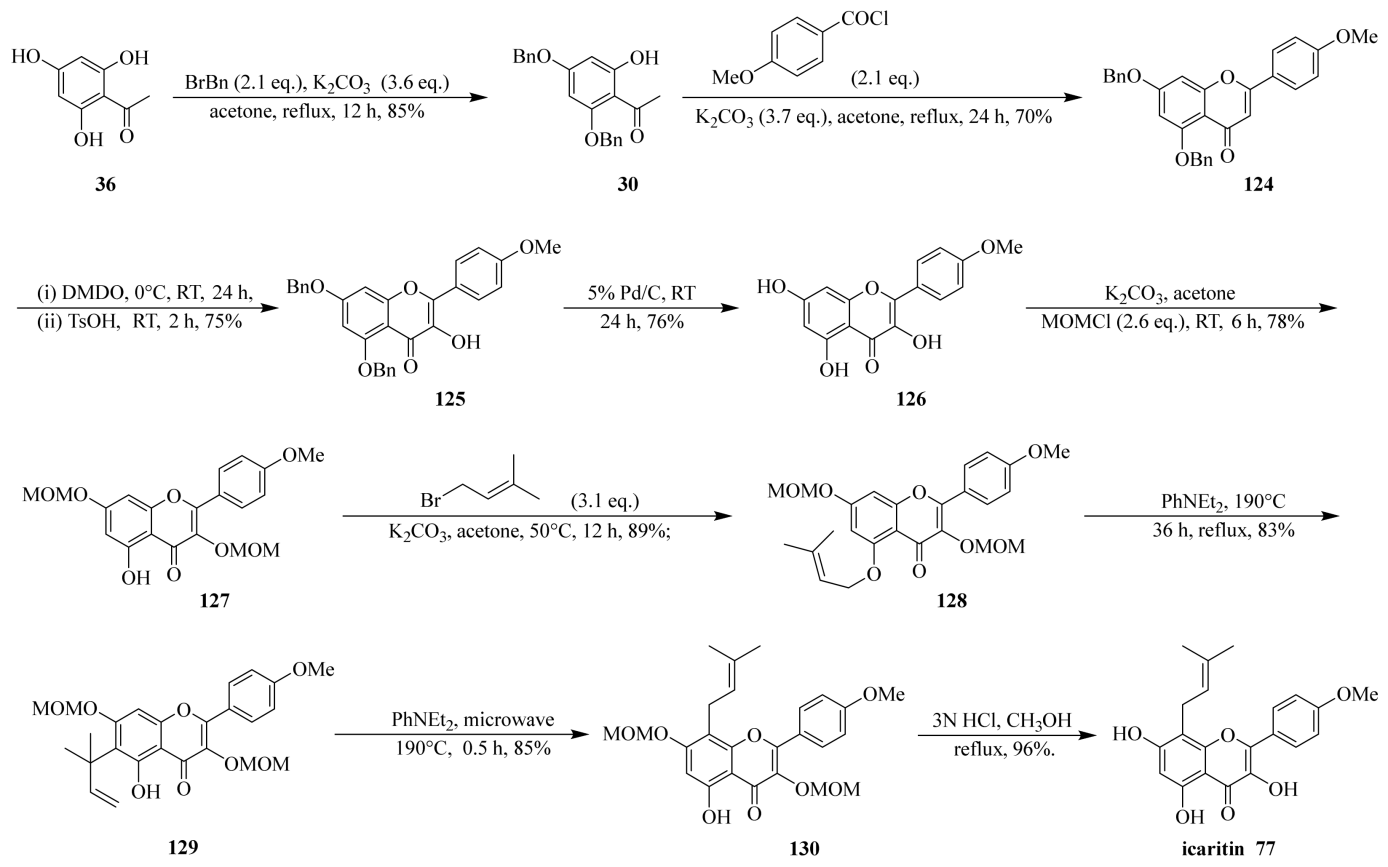
Synthetic route of icaritin **77** via DMDO oxidative reaction.

In 2014, Nguyen et al. [72] reported the total synthesis of icaritin **77** via microwave-assisted Claisen rearrangement. Starting from 2,4,6-trihydroxyacetophenone **36**, selective benzylation afforded **30**. Reaction with 4-methoxybenzoyl chloride gave the aryl ester, which underwent Baker–Venkataraman rearrangement and dehydrative cyclization to afford flavone **124**. Oxidation of flavone **124** with DMDO at low temperature, followed by acid-catalyzed rearrangement, furnished flavonol **125**. Hydrogenolysis of **125** over Pd/C removed the benzyl groups to give **126**. Selective O-methoxymethylation of **126** with MOMCl in dry acetone gave **127**. O-prenylation of the free 5-hydroxyl group of **127** using 3,3-dimethylallyl bromide as the electrophile gave **128**. Subsequently, microwave-assisted Claisen rearrangement of **128** in N,N-diethylaniline regioselectively afforded the *p*-prenylated product **130**, whereas conventional heating gave the ortho-isomer. Finally, acid-catalyzed deprotection of the MOM groups in **130** furnished icaritin **77** (Scheme 25). In this route, DMDO oxidation converted flavone to flavonol in 75% yield with complete C3 regioselectivity. Notably, DMDO serves as a mild, metal-free oxidant under neutral conditions, avoiding traditional harsh methods. However, the in situ generation of DMDO from oxone under controlled low temperature adds operational complexity and limits scalability.

#### 2.5.3. Synthesis of Houttuynoid A **138**

**Scheme 26:**
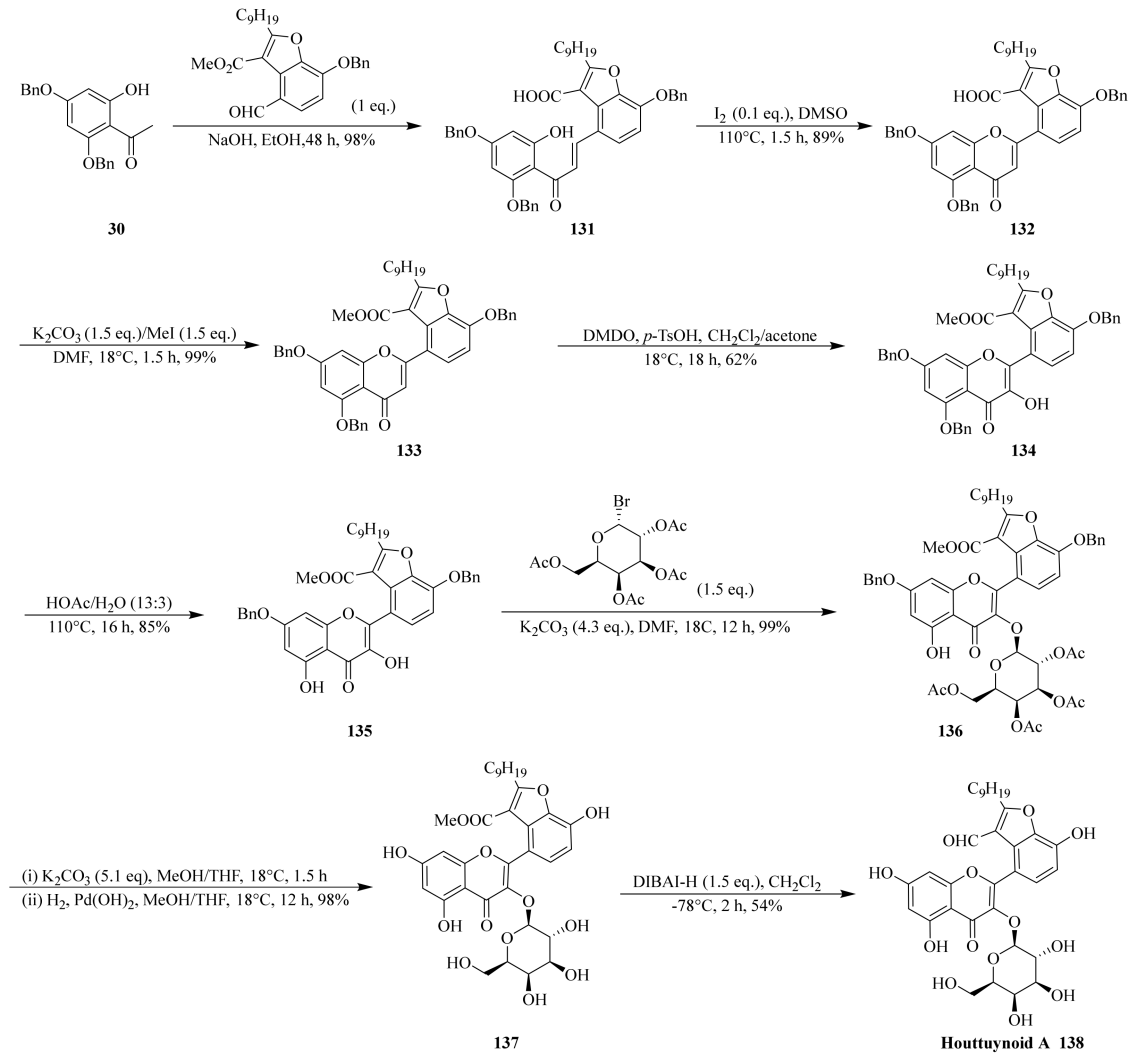
Synthetic route of Houttuynoid A **138**.

In 2018, Jian et al. [73] reported the first total synthesis of Houttuynoid A **138**, the most potent antiviral member of the Houttuynoid family. The synthesis commenced with a Claisen–Schmidt condensation between **30** and benzofuran aldehyde, using NaOH as a base, affording chalcone **131**. Subsequent I_2_-catalyzed oxa-Michael addition and oxidative cyclization of **131** in DMSO at 110 °C furnished **132**. Methylation of the C2′′ hydroxycarbonyl group in **132** afforded ester **133**, which was then subjected to hydroxylation at C3 using in situ generated DMDO to deliver flavonol **134**. Selective removal of the C5 benzyl group under acidic conditions allowed glycosylation with a galactosyl donor to yield **136**. Finally, deprotection and reduction afforded Houttuynoid A **138** (Scheme 26). The DMDO oxidation represents a key breakthrough, enabling C3 hydroxylation in 62% yield and overcoming a major obstacle in previous Houttuynoid B synthesis. The reaction features mild conditions, high selectivity, and good protecting-group compatibility. However, it suffers from poor atom economy, a prolonged reaction time of 32 h, and suboptimal yield, making it the rate-limiting step of the nine-step sequence. Despite these limitations, the method remains viable for laboratory-scale preparation.

Beyond this specific application, the broader utility of DMDO in flavonoid chemistry is constrained by its instability, limited storage time, and a demanding preparation [74]. To address these challenges, Wu et al. [75] introduced an intermittent addition of oxone aqueous solution to carefully control reaction temperature, addition rate, and duration. Through parameter optimization, the oxidation of flavones to flavonols resulted in over 95% yield with minimal by-product formation, aligning with green chemistry principles. Despite these advances, the process remains operationally cumbersome due to the thermal and chemical instability of the epoxidation products. Furthermore, DMDO-mediated oxidations display marked selectivity toward flavonoid substituent patterns, often leading to mixtures of isomers or side products that limit substrate scope and synthetic utility [76].

To provide a clear comparison of the total synthesis methods for the five polyhydroxy flavonols and their derivatives described in this review, Table 2 summarizes the key reaction conditions, protection/deprotection requirements, yields, scalability, advantages and limitations for each method.

## 3. Biological Activity

Flavonols are important plant secondary metabolites that exhibit a broad spectrum of biological activities, including antioxidant, anticancer, anti-inflammatory, antiviral, antidiabetic, and hepatoprotective effects. Detailed biological activities, mechanisms of action, and key references are summarized in Table 3.

**Figure 2:**
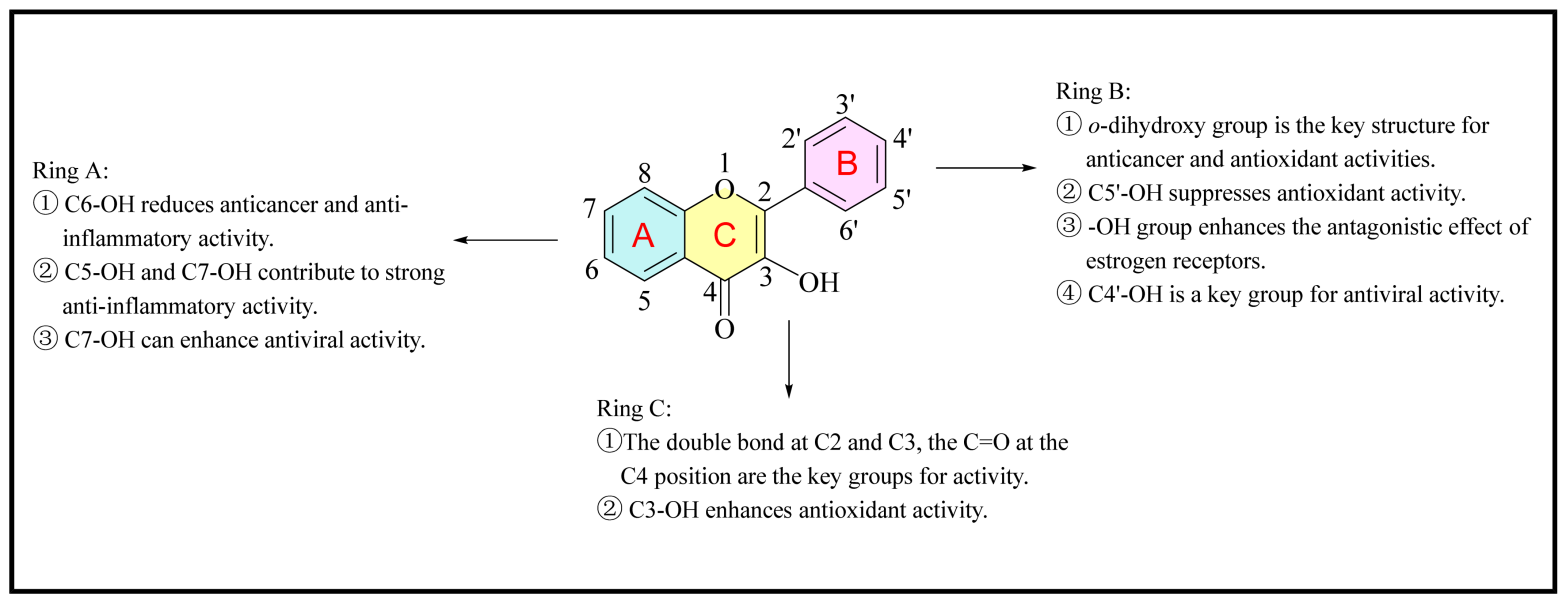
Structure–activity relationship diagram of polyhydroxy flavonols.

Flavonols are a widely distributed class of natural bioactive compounds in plants. Their biological activity is closely related to specific structural features, including the number and substitution pattern of hydroxyl groups, the presence of conjugated double bonds, and the overall molecular planarity. These structural attributes are key determinants of their pharmacological properties. Moreover, targeted structural modifications can profoundly influence both activity and bioavailability. Investigating these SARs provides fundamental insights into their mechanisms of action and enables the rational design of analogs with improved therapeutic profiles. This review systematically summarizes the current understanding of SARs of flavonols, offering a valuable framework to guide future research and potential applications (Figure 2). According to SAR analysis, the 6-OH group of ring A may reduce anticancer potential and anti-inflammatory activity by causing steric hindrance or affecting molecular planarity. Conversely, the simultaneous presence of hydroxyl groups at the C5 and C7 positions confers strong anti-inflammatory activity [90]. For example, gossypetin and galangin exhibit potent anti-inflammatory activity. Notably, the 7-OH group is essential for antiviral activity. Introducing alkyl chains of varying lengths at this position not only enhances the inhibitory activity against SARS-CoV-2 3CLpro but also improves cell membrane permeability [24]. The ortho-dihydroxyl group on the B ring is a key structural feature for anticancer and antioxidant activity [91]. For example, quercetin and fisetin contain a 3′,4′-ortho-dihydroxyl structure on the B ring and exhibit strong antioxidant activity due to the formation of stable semiquinone radicals following the reaction of the ortho-dihydroxyl group with free radicals. If a 5′-OH group is present in the B ring, antioxidant activity is reduced. For example, myricetin, which contains a 5′-OH group, exhibits lower activity than quercetin, which lacks a 5′-OH group. Additionally, the 4′-OH group is a key moiety for antiviral activity, as it acts as a hydrogen bond donor, forming hydrogen bonds with critical amino acid residues at the active site of viral proteases to enhance binding affinity [92]. For example, myricetin exhibits broad-spectrum antiviral activity against various viruses, including HIV, SARS-CoV-2, and EBOV. The C2=C3 double bond and the C4 carbonyl group in the C ring are essential structural features for biological activity [90]. Most active compounds retain these two functional groups, which jointly maintain the molecular conjugated system and planarity, thereby facilitating binding to the target. Furthermore, the 3-OH group on the C ring can form a conjugated system with the C2=C3 double bond to stabilize free radicals, thereby enhancing antioxidant activity and exerting a synergistic effect on anticancer activity [93].

## 4. Conclusions

Flavonols have attracted considerable research interest owing to their distinctive chemical structures and extensive biological activities. These compounds exhibit a broad spectrum of pharmacological effects, including antioxidant, anti-inflammatory, anticancer, and antiviral properties, making them highly valuable for medicine and functional food applications. In recent years, classical synthetic methods such as the Baker–Venkataraman rearrangement and the AFO reaction have been widely employed. Advances in reaction design, including one-pot protocols and green catalysts, have significantly improved synthetic efficiency. Nevertheless, the synthesis of flavonols still faces challenges such as multistep sequences, moderate yields, limited regioselectivity, and issues related to atom economy and rate-limiting steps.

Flavonols can be obtained through either natural extraction or chemical synthesis, with each method complementing the other in terms of application. Natural extraction is simple and cost-effective, suitable for obtaining high-content and simple-structured flavonols from plants. However, it is limited by factors such as plant resources, seasonality, and geographical constraints. It also faces sustainability challenges and struggles to produce rare monomers and flavonol derivatives. In contrast, chemical synthesis allows for precise control over product structure, and offers high yields and strong scalability, making it suitable for the synthesis of novel flavonols and SAR studies. However, for flavonols with multiple hydroxyl groups or complex glycosidic modifications, the chemical synthesis process is cumbersome and costly, making natural extraction more appropriate.

In addition to traditional preparation methods, biosynthesis represents a cutting-edge direction for the green production of flavonols. This method utilizes genetically engineered microorganisms to synthesize target products in fermenters, with heterologous biosynthesis serving as the mainstream technology. It features renewable resources, mild reaction conditions, high regioselectivity and stereoselectivity, and environmental friendliness, effectively addressing the shortcomings of chemical synthesis, such as high pollution and numerous side reactions. However, this technology currently faces challenges including long production cycles and insufficient yields for industrial applications. Looking ahead, a combined chemical–enzymatic approach could be adopted, utilizing chemical synthesis to construct the core skeleton of flavonols, followed by enzyme-catalyzed modification at specific sites. This would strike a balance between synthetic efficiency and regioselectivity, representing a promising future direction for this field.

Future efforts should focus on developing more efficient and selective synthetic methodologies, optimizing synthesis processes, and conducting deeper investigations into structure–activity relationships. Such studies will enhance the clinical translatability and practical utility of flavonols.

## Figures and Tables

**Table 1:** Common polyhydroxy flavonols.

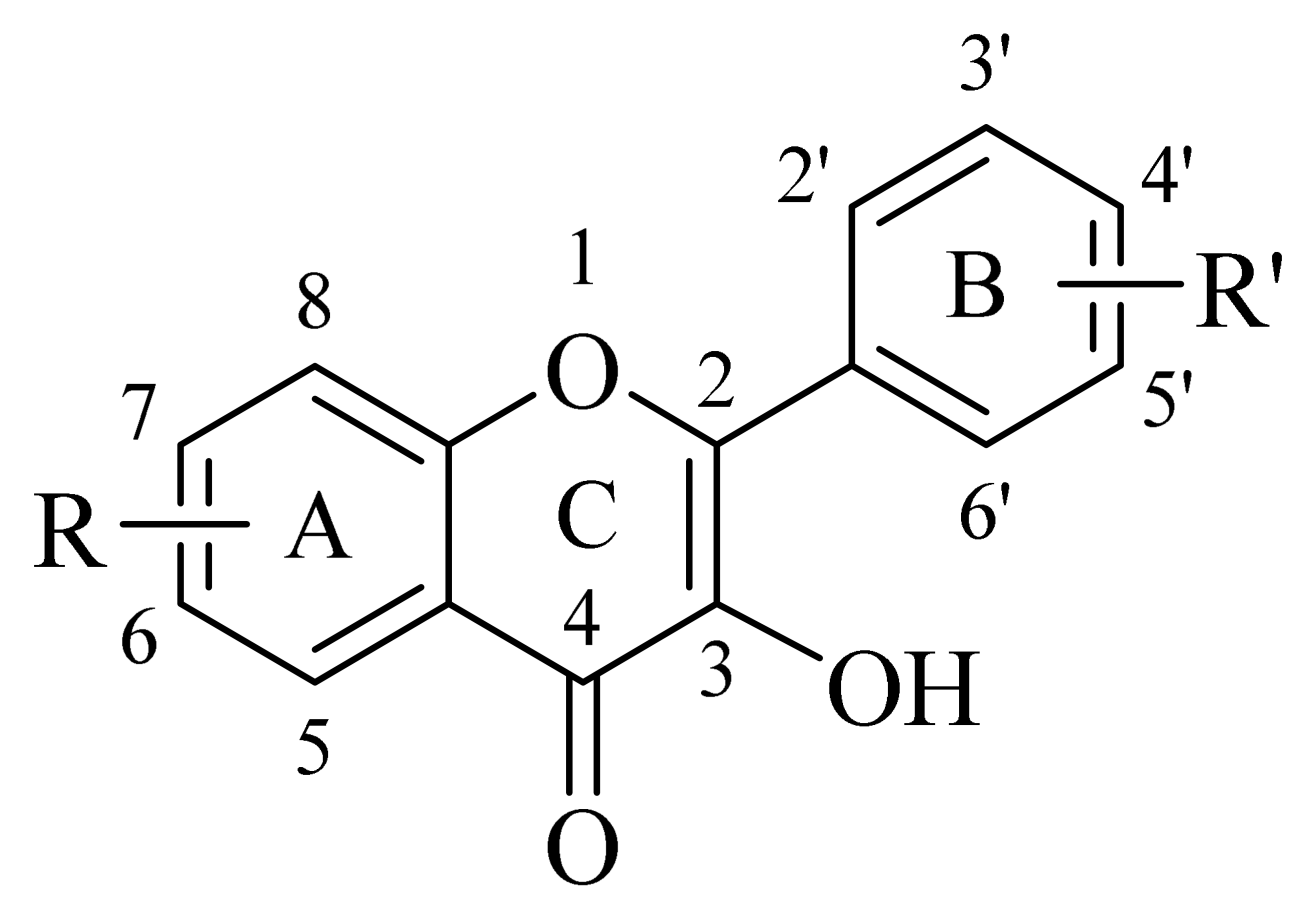								
Compound	R^5^	R^6^	R^7^	R^8^	R^2′^	R^3′^	R^4′^	R^5′^
kaempferol	OH	H	OH	H	H	H	OH	H
myricetin	OH	H	OH	H	H	OH	OH	OH
quercetin	OH	H	OH	H	H	H	OH	OH
galangin	OH	H	OH	H	H	H	H	H
morin	OH	H	OH	H	OH	H	OH	H
gossypetin	OH	H	OH	OH	H	H	OH	OH
quercetagetin	OH	OH	OH	H	H	H	OH	OH
fisetin	H	H	OH	H	H	H	OH	OH

**Table 2:** Comparison of total synthesis methods for polyhydroxy flavonols.

Synthesis Methods	Key Reaction Conditions	Protection/Deprotection Requirements	Yields (Key Step)	Scalability	Advantages	Limitations
Auwers reaction	(1) alkaline rearrangement.	-	low	Not applicable.	(1) Applicable to flavonol synthesis.	(1) Stringent structural requirements for reactants. (2) Difficult preparation of dibromide intermediates with low yields.
Allan–Robinson reaction	(1) Aromatic anhydride to provide B-ring. (2) Base conditions for cyclization.	Protecting groups: CH_3_O-. Deprotection methods: BBr_3_.	about 53%	Moderate scalability for improved method.	(1) Streamlined one-pot operation. (2) Moderate yields.	(1) Formation of 3-aryloxyflavone by-products. (2) Poor atom economy.
Baker–Venkataraman rearrangement reaction	(1) Base conditions for rearrangement. (2) acid for cyclization.	Protecting groups: THPO-, MOMO-, CH_3_O-, BnO-, AcO-. Deprotection methods: TsOH/MeOH, HCl/MeOH, Py·HCl or BBr_3_, Pd/C, K_2_CO_3_/MeOH.	20–74%	Conventional: limited scalability. improved: scalable.	(1) Readily accessible starting materials. (2) Mild reaction conditions. (3) Broad substrate scope.	(1) Tedious reaction process. (2) Moderate yields.
AFO reaction	(1) Mild basic conditions. (2) H_2_O_2_ or air as oxidant.		30–98%	Wide range of compatible substrates.	(1) Mild reaction conditions. (2) Broad substrate scope. (3) Streamlined one-pot operation. (4) Green chemistry compatibility. (5) Good scalability.	(1) Formation of aurone by-products. (2) Moderate yields for certain substituted substrates.
DMDO oxidative reaction	(1) DMDO as oxidant.		62–75%	Limited by DMDO instability and demanding preparation	(1) Good yields under optimized conditions. (2) Minimal by-product formation. (3) High regional selectivity at the C3 position. (4) Green chemistry compatibility.	(1) Instability and limited storage time of DMDO. (2) Demanding preparation of DMDO. (3) Operationally cumbersome process. (4) Limited substrate scope and synthetic utility.

**Table 3:** Summary of the biological activities of polyhydroxy flavonols.

Activity		Compounds	Chemical Structure	Mechanism of Action	Refs.
Antioxidant		Fisetin	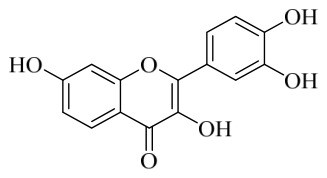	Activates Nrf2/ARE pathway. Upregulates HO-1.	[11,12,77]
		kaempferol	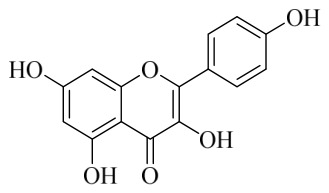		
Anticancer	Breast Cancer	Myricetin	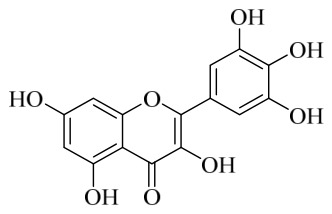	Induces ROS-dependent mitochondrial apoptosis. Activates the BRCA1-GADD45 pathway.	[18,78]
		Quercetin	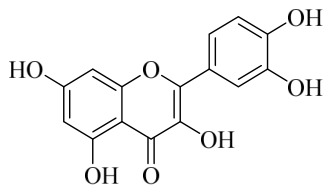	Upregulates Bax and Caspase-3. Downregulates Bcl2.	[79]
	Ovarian Cancer	Quercetin	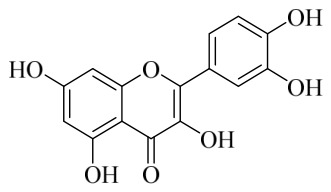	Inhibits CDK/cyclin expression. Activates Bax/Bcl-2-caspase pathway.	[80,81]
		Morin	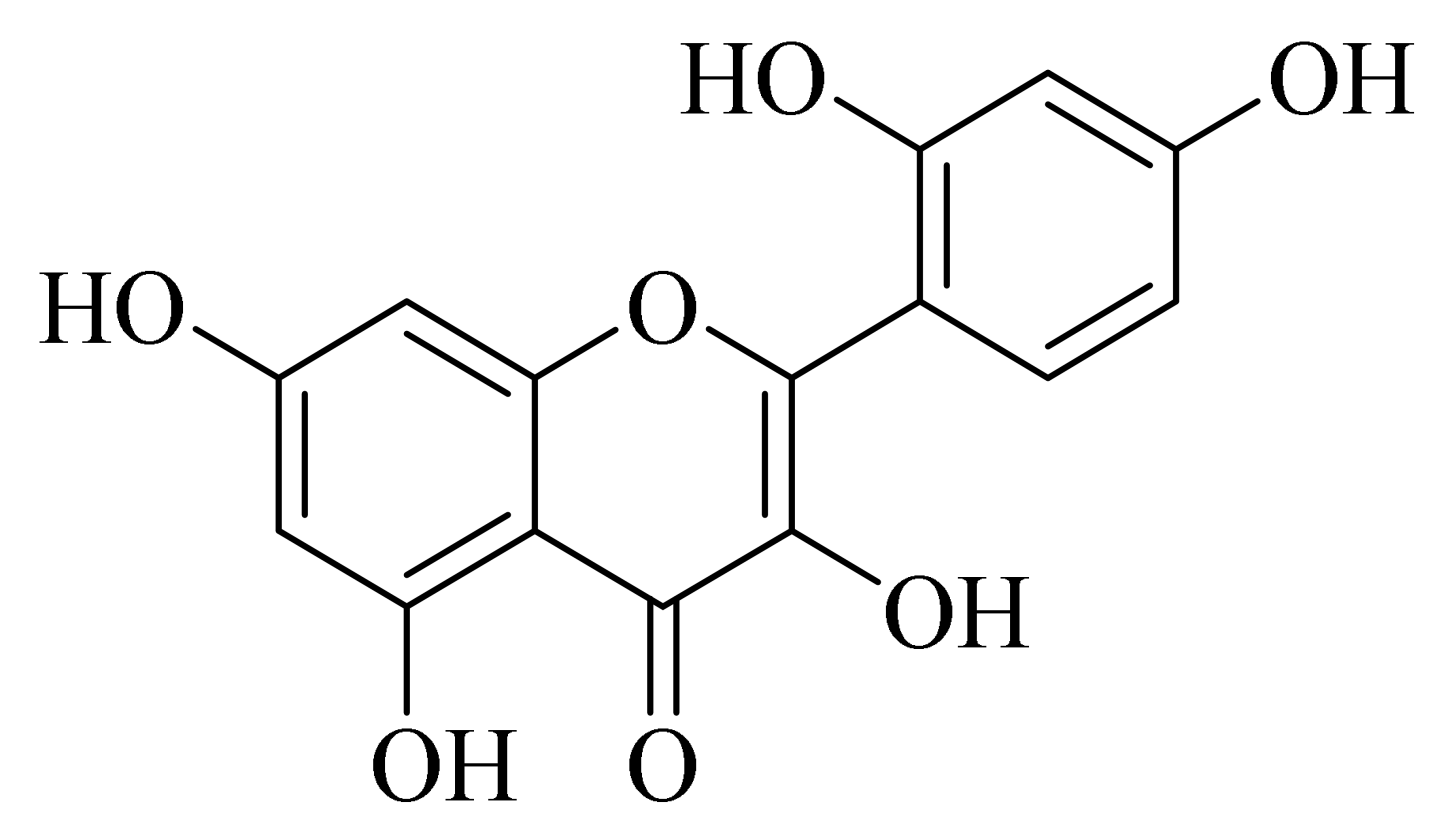	Inhibits NF-κB pathway.	[82]
	Skin Cancer	Fisetin	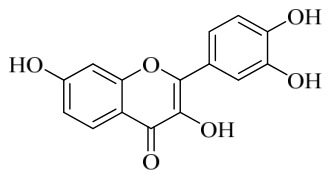	Inhibits PI3K pathway. Downregulates TNF-α, IL-1β, IL-2.	[83]
Anti-inflammatory		Gossypetin	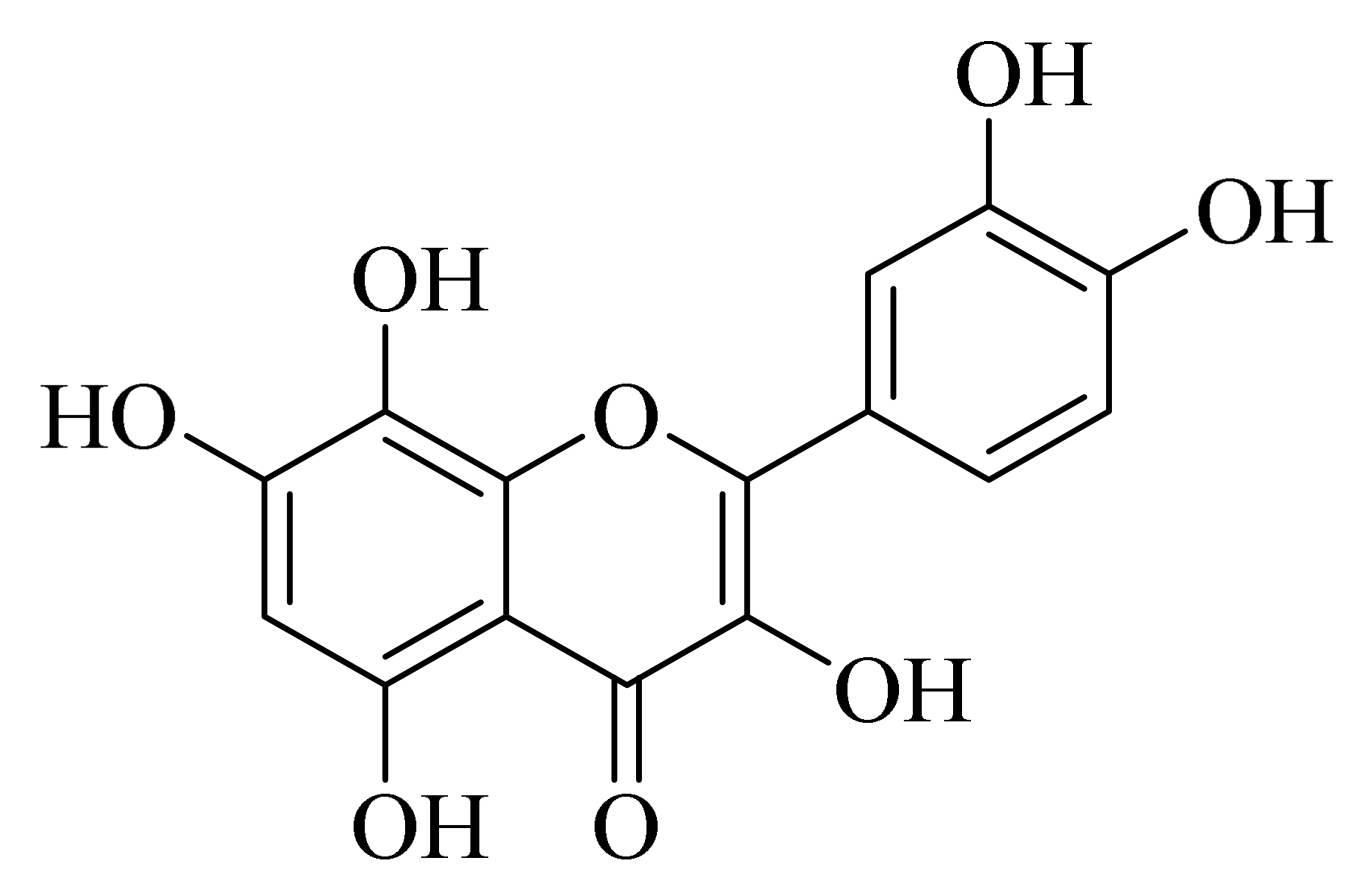	Inhibits MKK3/6-p38 MAPK signaling pathway. Downregulates IL-1α, IL-1β, TNF-α expression.	[84,85]
		Galangin	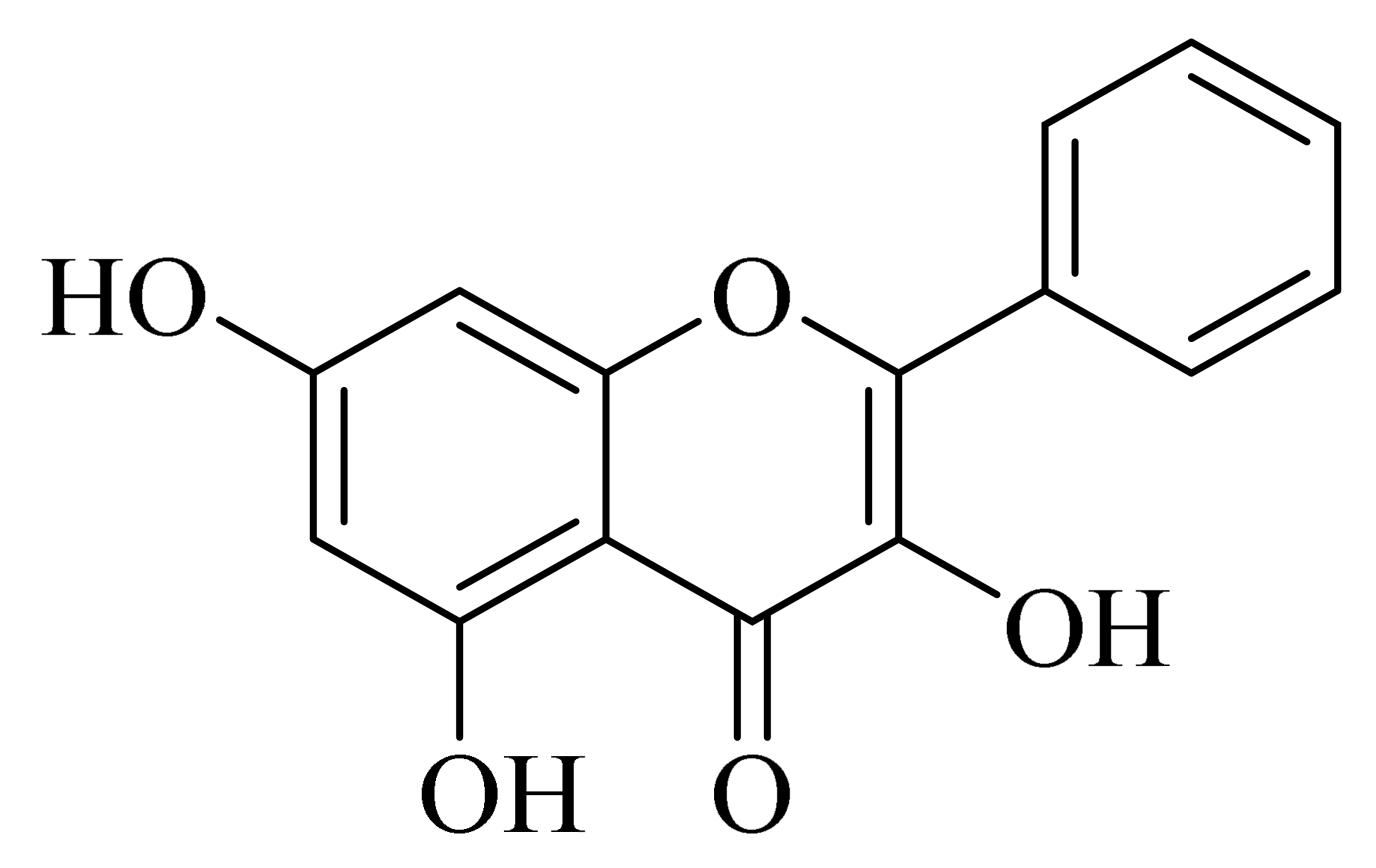	Inhibits NF-κB signaling pathway. Inhibits iNOS, COX-2 expression.	[86,87]
Antiviral		Myricetin	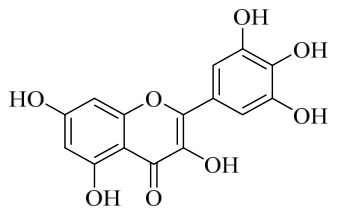	Inhibit 3CLpro/M pro. Inhibits RIPK1/NF-κB signaling pathway.	[88,89]

## Data Availability

No new data were created or analyzed in this study.

## Abbreviations

The following abbreviations are used in this manuscript:

EA	Ethyl Acetate
TEA	Triethylamine
BBr_3_	Boron Tribromide
LiHMDS	Lithium bis(trimethylsilyl)amide
DBU	1,8-Diazabicyclo [5.4.0]undec-7-ene
*p*-TsOH	p-Toluenesulfonic Acid
TFA	Trifluoroacetic acid
PPA	Polyphosphoric Acid
MOM	methoxymethyl ether
DHP	3,4-Dihydro-2H-pyran
PPTS	Pyridinium p-Toluenesulfonate
EDC	1-(3-Dimethylaminopropyl)-3-ethylcarbodiimide (hydrochloride)
DMAP	4-Dimethylaminopyridine
TBAB	Tetrabutylammonium bromide
DMF	N, N-Dimethylformamide
DEG	Diethylene glycol
LDA	Lithium diisopropylamide
TMSCl	Chlorotrimethylsilane
THF	Tetrahydrofuran
m-CPBA	meta-Chloroperoxybenzoic acid
BF_3_·OEt_2_	Boron trifluoride etherate
PTT	phenyltrimethylammonium tribromide
Pd(PPh_3_)_4_	Tetrakis(triphenylphosphine)palladium
*i-*PrOH	Isopropyl Alcohol
Py	pyridine
DEP	diethyl phosphite
DMS	dimethyl sulfate
Bi(OTf)_3_	Bismuth(III) trifluoromethanesulfonate
NMP	N-Methyl-2-pyrrolidone
TsOMe	Methyl p-toluenesulfonate
TBHP	Tert-Butyl hydroperoxide
DMDO	Dimethyldioxirane
Oxone	potassium peroxymonosulfate
DFT	density functional theory
DMSO	Dimethyl Sulfoxide
MOMCl	Chloromethyl methyl ether
PhNEt_2_	N, N-Diethylaniline
DIBAL-H	Diisobutylaluminium hydride
SAR	Structure–activity relationship
